# Assessing the spatiotemporal distribution of bufonid herpesvirus 1 (BfHV1) in Europe

**DOI:** 10.1038/s41598-025-06460-5

**Published:** 2025-06-25

**Authors:** Philipp Böning, Tobias Hildwein, Viktoria Ferner, Jonas Henn, Eva Kappe, Jesse Erens, Benjamin Lamp, Tobias Eisenberg, Amadeus Plewnia

**Affiliations:** 1 Biogeography Department, Trier University, Universitätsring 15, 54296 Trier, Germany; 2 Geobotany Department, Trier University, Universitätsring 15, 54296 Trier, Germany; 3 Department of pathology, Bavarian Animal Health Service, Senator- Gerauer-Str. 23, 85586 Poing-Grub, Germany; 4 Institute of Virology, Justus Liebig University Giessen, Schubertstrasse 81 (BFS), 35392 Giessen, Germany; 5 Hessian State Laboratory, Schubertstraße 60, 35392 Giessen, Germany

**Keywords:** Wildlife-only disease, Bufonids, *Batravirus*, Citizen science, Amphibian crisis, Herpetology, Biogeography, Ecological epidemiology

## Abstract

Adverting biodiversity loss is one of the most urgent challenges of our time. The ongoing amphibian extinction crisis is the result of a multitude of factors, with emerging infectious diseases having played a key role. While extensive contributions have been made to study chytrid fungi and ranaviruses in the last two decades, other amphibian pathogens have remained largely unstudied. Here, we evaluated the spatiotemporal distribution of Bufonid herpesvirus 1 (BfHV1) in Europe, a pathogen capable infecting true toads (family Bufonidae). Using molecular detection and histology, we identified seven new BfHV1 positive sites in Germany and a first record for Luxembourg. Phylogenetic analysis of samples from these sites revealed a monophyletic cluster with the known BfHV1 reference sequences. Through additional systematic examination of photographic records from citizen scientists, we identified 229 BfHV1 cases (62 positive, 167 suspicious) in the genus *Bufo* (*B. bufo*, *B. spinosus*), with suspicious cases being widespread across Europe and dating back until at least 2007. As such, this first continental assessment suggests that BfHV1 has been rather overlooked than being recently emerging. Yet, in view of increasing observations of population declines in bufonids across Europe, additional research is warranted to assess its effects on amphibian populations.

**Supplementary Information:**

The online version contains supplementary material available at 10.1038/s41598-025-06460-5.

## Introduction

The world´s amphibians are experiencing unprecedented declines, leaving them as the most imperilled vertebrate class today^1,2^. This ongoing crisis is primarily driven by habitat loss, climate change, and emerging infectious diseases^3^. Among the latter, epidemics of the amphibian chytrid fungi *Batrachochytrium dendrobatidis* (*Bd*) and *B. salamandrivorans* (*Bsal*), along with Ranaviruses (*Rv*) have had devastating effects on amphibian populations^4–11^. Numerous other pathogens have been linked to amphibian mortality events [e.g.^12–14^]. However, their impact on wild populations, origins, and spatiotemporal distribution remain poorly understood. Disease introductions, especially in combination with other existing threats, can nevertheless cause steep declines even in locally abundant species [e.g.^15,16^]. Mitigating the effects of diseases on wild populations remains highly challenging but has most potential during early invasion stages [e.g.^17,18^]. This is further aggravated by drastic resource limitations and a lack of attention from authorities in monitoring and preventing further spread of wildlife-only diseases^19^. Drawing attention to newly discovered and under-researched amphibian pathogens may therefore prove crucial to avert further disease-driven declines and extinctions.

Amphibian herpesviruses in the family Alloherpesviridae exemplify understudied and potentially overlooked pathogens^20,21^. Although being known for almost a century^22^, only recent studies have begun uncovering the diversity and distribution of amphibian herpesviruses. Several amphibian herpesviruses have been identified from Bufonidae, Pelobatidae and Ranidae and might be qualified as emerging infectious diseases (EID)^21,23–27^. Infections with a virus, tentatively named bufonid herpesvirus 1 (BfHV1), have so far only been reported in populations of the common toad (*Bufo bufo*) in Switzerland and two federal states of Germany (Hesse and Thuringia)^25,27^. BfHV1 infection manifests as raised, multifocal to confluent patches of dark, thickened skin and can lead to mortality in the wild^25,27^. BfHV1 was discovered only recently in 2014^25^, leaving it unclear whether the pathogen is emerging in Europe or has long been endemic but overlooked. Solving this question will be of particular importance in view of widespread declines of European toads. Across Europe, enigmatic declines in bufonids have been reported repeatedly, underlining the necessity to study pathogenic agents and their potential role in the observed host declines to avert further biodiversity loss^28–30^. Combining molecular and histologic examination of symptomatic specimens with systematic examination of citizen scientists’ photographic records of the characteristic clinical signs, we suggest a yet overlooked broad distribution of BfHV1 across Europe most likely pre-dating the pathogen’s description.

## Methods

### Molecular detection

During recent amphibian disease screenings in Germany, particularly for *Bsal*^11^, common toads were opportunistically included, especially when found with skin alterations as described above. Samples comprised dry skin swabs (Medical Wire & Equipment, Corsham) taken by softly rubbing over the entire body of *B. bufo* specimens or epidermal remnants from their skin sheddings. Body parts with macroscopically visible skin alterations were also included. Each animal was continuously swabbed with at least 50 strokes comparable to sample protocols of other amphibian skin diseases^31^. We extracted DNA using the Blood & Tissue kit (Qiagen, Hilden) following a modified protocol detailed by^32^. We targeted the BfHV1 specific diagnostic marker developed by^25^ using end-point PCR and visualization via gel electrophoresis. For each locality with successful amplification, we selected one PCR product for subsequent Sanger dideoxy sequencing. Amplicons were purified using the High Pure PCR purification kit (Roche, Basel) and shipped to Macrogene Europe for sequencing. Sequence reads were processed in Geneious Prime version 2025.0.3 using default settings for trimming and consensus generation. We mapped all sequences to a local copy of NCBI`s nucleotide database using Blastn version 2.5.0 + for sequence and taxonomic identification. We further employed RAxML^33^ implemented in Geneious Prime for phylogenetic reconstruction using the LG protein model with rapid bootstrapping (10.000 replicates) and a random starting tree. We included available sequences from GenBank and two ranid herpesvirus sequences (Ranid HV1 [RaHV1; now *Batravirus ranidallo1*; accession number YP656727.1], RaHV2 [now *Batravirus ranidallo2*; accession number ABG25576.1]) as outgroups following the phylogeny of^25^. Then, we created a consensus tree in Geneious Prime (support threshold: 50%; burn-in: 1,000) and visualized the result in FigTree (version 1.4.4).

### Histology

We examined one specimen of *Bufo bufo* with macroscopic BfHV1-like skin lesions from Luxembourg (Niederanven, Sennigerberg, found on 31.03.2021 and provided by R. Stassen, Biota.lu) with histology as we were unable to extract genomic DNA due to formalin preservation of the specimen. Skin biopsies from throat, ventral thorax and abdomen were fixed in 10% buffered formalin. Following processing for paraffin embedding, 4-µm-thick sections were cut and stained with haematoxylin and eosin (HE).

### Photographic detection and spatial analysis

To explore the spatial and temporal prevalence of BfHV1 infection across Europe, we analysed manually referenced photographs of central European true toad species (*B. bufo*,* B. spinosus*,* Bufotes viridis* and *Epidalea calamita*) submitted by citizen scientists to the Global Biodiversity Information Facility (GBIF) online database^34–37^. We also reviewed photographic records on the citizen science platform Observation.org^38–41^ to address delays in data uploads to GBIF. Additionally, we compiled photographs submitted directly to the authors. For systematic screening, we filtered occurrence data in GBIF by species name, basis of record (“human observation” and “observation”), continent (“Europe”), and publisher (all, except “Observation.org”). In Observation.org, we searched for each of the three species separately, including all observation data with a photographic documentation. In both databases, we included photos uploaded until December 2024. These records (*B. bufo*: 152,010; *B. spinosus*: 13,515; *B. viridis*: 9,598; *E. calamita*: 12,557) were visually examined for the presence of any kind of skin alterations indicating a potential skin disease (e.g. multifocal lesions, round to irregularly shaped ulcerations with brown margins and up to 10 mm in diameter, dark/black spots and patches that appeared superficially thickened, crusty, cauliflower-like and slightly elevated above normal skin level, wounds excluding scars; see as well^28^). Subsequently, these were independently scored by at least three authors to identify typical clinical signs consistent with BfHV1 (see Supplementary Table S1, Supplementary Figure S3-S4), following^25^ or to assign the observed lesion to other clinical pictures. Records showing clinically suspicious signs of BfHV1, agreed upon by all examining authors, were extracted as suspicious cases. For geographic visualization of our dataset, we used ArcGis Pro (version 3.1.0)^42^. In detail, we computed a heatmap for suspicious records (i.e. extracted from photo-database screening and photos sent to authors, see Supplementary Table S1) with the “kernel density” tool (raster size = 0,005 decimal degrees; search radius = 1 decimal degree; method = planar) after deleting records with identical coordinates. For comparison, we computed a heatmap with same settings using all georeferenced records of *B. bufo* and *B. spinosus* until December 2024 available from GBIF (same filters as above but including data from Observation.org)^43^. We used R (version 4.4.2) for data visualization (package “ggplot2”). In detail, we used all currently known records and suspicious cases from the literature and from this study to show the temporal extent as well as the monthly distribution of the disease in Europe. For the latter, we show occurence data per month derived from all years.

## Results

### Molecular detection

23 of the 45 samples from seven German localities showed a distinctive PCR amplicon in gel electrophoresis (Supplementary Fig. S1). Sanger sequencing confirmed BfHV1 presence in all samples sequenced with 100% query coverage and over 99% sequence identity with the BfHV1 reference sequence (NC_040681; Supplementary Table S2). The phylogenetic analyses grouped all sequences into a monophyletic clade with 100% consensus support, including our new and known BfHV1 sequences, closely related to RaHV1 as shown in prior studies (Supplementary Fig. S2)^25^. The positive tested specimens showed characteristic large multifocal or confluent skin lesions with crusty, cauliflower-like appearance or, in some specimens, small round skin lesions with ulcerations surrounded by a dark brown margin (Supplementary Fig. S3, S4). These lesions are of similar appearance to those reported for *Bsal* infection in salamanders with visual examination^44^. However, subsequent qPCR diagnostics^45^ confirmed the absence of *Bsal* in all samples.

### Histology

The patho-histological examinations revealed epidermis of normal thickness in the peripheral lesions. Occasionally, exocytosis of heterophil granulocytes into the epidermis was present. Within the lesions a moderate, irregular epidermal hyperplasia accompanied by rete ridge formation, mild spongiosis, and focal exocytosis of granulocytes was present. The epidermis exhibited hyperkeratosis with both orthokeratotic and parakeratotic areas, and pigment deposits localized in the stratum superficiale. Further, intranuclear inclusion bodies were suspected in degenerated keratinocytes. These nuclei showed eosinophilic to amphophilic inclusions with margination of chromatin (Supplementary Fig. S5).

### Photographic documentation and detection

**Fig. 1:**
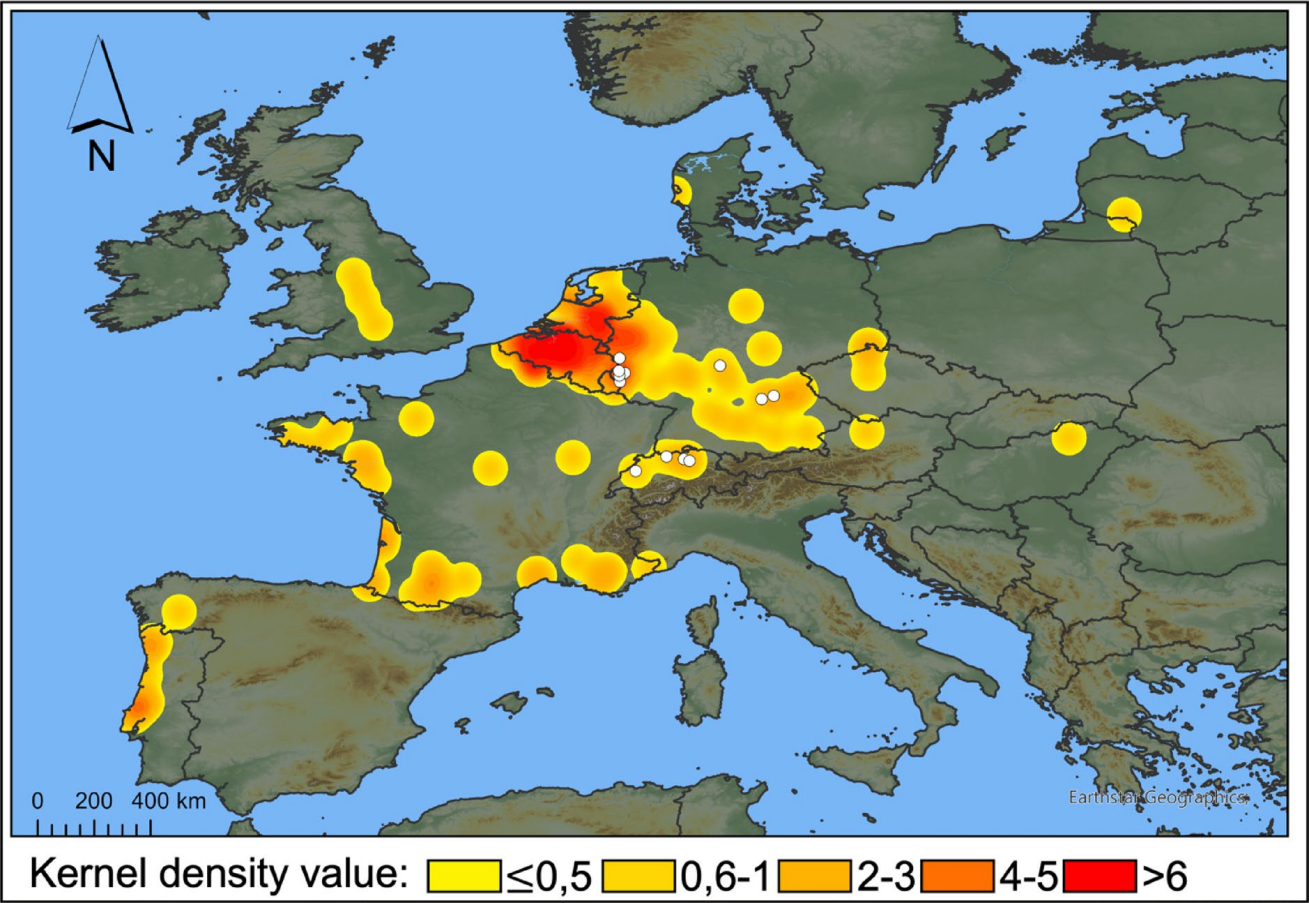
Spatial extent of Bufonid herpesvirus 1 records across Europe (WGS 1984). White dots correspond to molecular and histological records. The heatmap shows the distribution of suspicious cases derived from referenced photographs.

**Fig. 2:**
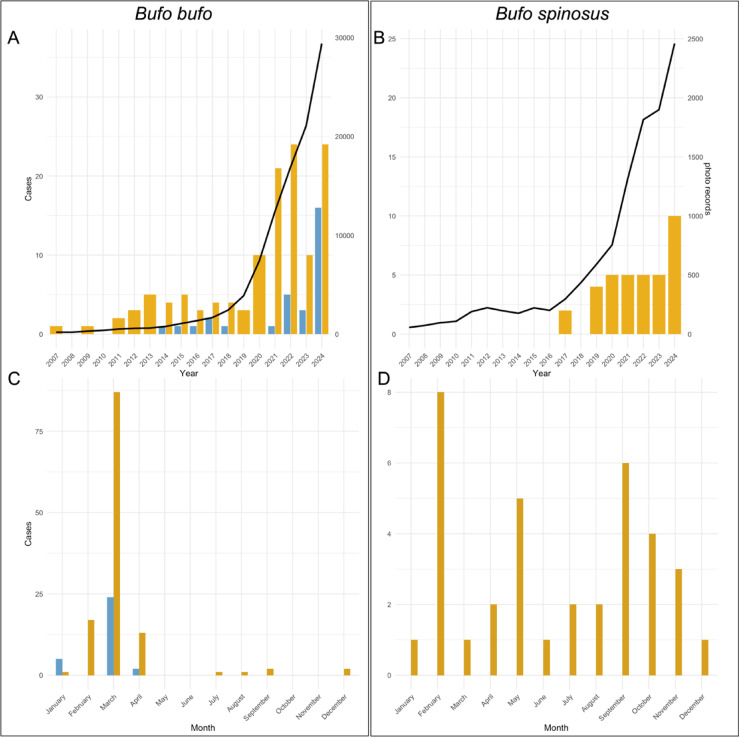
Temporal extent of Bufonid herpesvirus 1 records in Europe. Distribution of positive (derived from molecular diagnostics, histology and literature, blue bars) and suspicious (photographs of clinical signs, yellow bars) records between 2007 and 2024 in (**A**) *Bufo bufo* and (**B**) *B. spinosus;* over the course of the year summarizing all years (2007–2024) for (**C**) *B. bufo* and (**D**) *B. spinosus*. Trendlines in black show all photographs of the respective species available from GBIF (*B. bufo*: 10.15468/dl.8ahbw8; *B. spinosus*: 10.15468/dl.hthn5e).

In total (GBIF, Observation.org, photos received), we identified 167 cases in two species from 158 localities as “suspicious”, spanning 14 European countries from 2007 to 2024 (including the UK, Fig. 1, Supplementary Table S1). Of these, 125 cases belong to *B. bufo*, having a density centre in the three countries Belgium, Germany and the Netherlands (Fig. 1), and 33 to *B. spinosus* (Supplementary Table S1). Suspicious observations largely coincide with the spatial distribution of all records of both species (Supplementary Fig. S6). Further, suspicious cases identified before the scientific description of BfHV1 in 2014, were found in those three countries (Supplementary Table S1). Overall, the number of suspicious cases found in online databases increased over the last five years in both species (*B. bufo* > 10 individuals; *B. spinosus* ≥ 5 individuals) at a similar rate to the increase in number of photos uploaded (Fig. 2A, B). Moreover, typical macroscopic BfHV1-like skin lesions in *B. bufo* were observed almost exclusively during the spawning season of the host between February and April (Fig. 2C). However, in *B. spinosus*, BfHV1 suspicious cases were identified in all months with slightly more cases in February and September (Fig. 2D). We identified additional skin anomalies in 103 cases in four toad species (*B. bufo*, *B. spinosus*, *Bufotes viridis*, *Epidalea calamita*), suggesting the presence of other skin diseases (Supplementary Table S1). Photo quality often limited the identification of macroscopic lesions. Therefore, we cannot state how many animals were free of BfHV1-like lesions. Additionally, we could not classify observed skin anomalies to typical clinical signs of BfHV1 in 14% of all skin artefacts detected (marked as “BfHV1 uncertain” in Supplementary Table S1).

## Discussion

For the first time, we compiled positive (*N* = 63; 25 this study, 38 from literature) and suspicious (*N* = 167; all this study) BfHV1 cases across Europe (Supplementary Table S1). Our results show that BfHV1 is more widespread and may infect more host species than previously believed, with records extending the pathogen’s distribution from currently known two to three and potentially up to 14 European countries (including the UK and Switzerland)^25,27,46^. Particularly worrying in this regard are the sharp declines reported for *B. bufo* populations across Europe^25,27–30,47,48^, potentially linked to diseases.

Photo-based identification has unravelled a potential new host species (*B. spinosus*) and predates the possible emergence of BfHV1 in Europe by seven years, with observations dating back to 2007 (Fig. 2)^25^. This, in line with the distribution and the occurrence of suspicious cases identified before 2014, indicates that BfHV1 has been missed broadly and is likely more widespread and abundant than previously thought. Further, the strong link between suspicious cases and the overall spatial reporting pattern by citizen scientists, suggests that additional occurrences remain overlooked to date (Supplementary Fig. S6). Therefore, it is very likely that the virus has been rather overlooked than was recently emerging in Europe.

BfHV1 occurrence in populations of *B. bufo* is mostly restricted to the spawning season^25,49^. The temporal pattern aligns with findings on the sister lineage RaHV1, which shows higher viral replication under cold temperatures as during the hosts spring migration^50^. For *B. spinosus*, sampling size is small, and no seasonality appears obvious. So far, it further remains to be tested if other reservoir species or the environment play a role in the seasonality of BfHV1 occurrence or if the pathogen persists without showing clinical signs in the host during the other seasons^51^.

The photo-based detection method in this study has notable limitations and can therefore only provide indications. First, prevalence as well as asymptomatic BfHV1 infections remain unknown. Additionally, photo quality influences the observer’s decision on how to categorize individual BfHV1 disease status. Hence, those constraints potentially lead to an underestimation of BfHV1 occurrence within populations and geographic distribution. Second, molecular diagnostics are essential for verification, as photo-based identification serves only as a preliminary tool for identifying suspicious cases or populations without definitive confirmation^46,52^. However, as diagnostic samples of the focal toad species across broad spatial and temporal scales are currently unavailable, we consider photo-based examination a powerful tool to guide future selection of sampling sites for molecular diagnostics and to identify hypothetical centres of BfHV1 infection in Europe.

The citizen science data identified Belgium and adjacent regions of the Netherlands and Germany as the hotspot of BfHV1 infections. An in-depth pathogeographic study is necessary to rule out if this region is indeed the endemic centre of BfHV1 in central Europe. However, we rather expect that this result is based on observation bias, as the records processed in this study are most dense in this region (Supplementary Fig. S6).

Sampling historic, formalin preserved specimens from museum collections with histology and ancient-DNA high-throughput sequencing approaches as well as assessing potential lineage diversity present the next steps towards delimiting the origin of BfHV1 [e.g.^53,54^]. Additionally, it will be crucial to assess a possible BfHV1 spillover to yet naïve regions.

The role in population declines as well as within-population disease dynamics remain unknown for all amphibian herpesviruses^49^. Therefore, multi-year studies alongside infection experiments are now of utmost importance^55^. This will contribute to understanding the impact of amphibian herpesviruses on different geographic and biological scales, helping to prevent further disease-driven amphibian losses.

## Electronic supplementary material

Below is the link to the electronic supplementary material.

Supplementary Material 1.

## Data Availability

All data is outlined in Supplementary Material 1. The Sequencing data generated during the current study is available on genebank (https://www.ncbi.nlm.nih.gov/genbank/) under the accession numbers PV005823-PV005829.
