# Supplementary material for: Assessing the spatiotemporal distribution of bufonid herpesvirus 1 (BfHV1) in Europe
Source: Sci Rep. 2025 Jun 25;15:20289. doi: 10.1038/s41598-025-06460-5 (PMC12198397; doi:10.1038/s41598-025-06460-5)
Supplement: Supplementary file 1 — Supplementary Material 1. [file 41598_2025_6460_MOESM1_ESM.pdf]

Assessing the spatiotemporal distribution of Bufonid herpesvirus 1 (BfHV1) in Europe

Philipp Böning, Tobias Hildwein, Viktoria Ferner, Jonas Henn, Eva Kappe, Jesse Erens,

Benjamin Lamp, Tobias Eisenberg, Amadeus Plewnia

Supplementary Material

Supplementary Table S1: Records of BfHV1 derived from molecular or histological examination as well as suspected cases based on photographs.

ID	Sample ID	Species	Date	Country	Locality	LAT	LON	record	photo	remarks	Histology	PCR	Sequence	Gen Bank Accsession	BfHV1 result	Reference 1
1	EK1 Kammerstein	*Bufo bufo*	14.03.24	Germany	Bavaria, Kammerstein	49,2915	10,9535	new state record	yes		no	pos.	no	N.A.	positive	this study
2	EK1a Kammerstein	*Bufo bufo*	14.03.24	Germany	Bavaria, Kammerstein	49,2915	10,9535	new state record	yes		no	pos.	no	N.A.	positive	this study
3	EK2 Kammerstein	*Bufo bufo*	14.03.24	Germany	Bavaria, Kammerstein	49,2915	10,9535	new state record	yes		no	pos.	yes	PV005828	positive	this study
4	EK3 Kammerstein	*Bufo bufo*	14.03.24	Germany	Bavaria, Kammerstein	49,2915	10,9535	new state record	yes		no	pos.	no	N.A.	positive	this study
5	EK4 Kammerstein	*Bufo bufo*	14.03.24	Germany	Bavaria, Kammerstein	49,2915	10,9535	new state record	yes		no	pos.	no	N.A.	positive	this study
6	EK1 WS	*Bufo bufo*	19.03.24	Germany	Bavaria, Altdorf, Winkelhaid	49,3937	11,3390	new state record	no		no	pos.	no	N.A.	positive	this study
7	EK2 WS	*Bufo bufo*	20.03.24	Germany	Bavaria, Altdorf, Winkelhaid	49,3937	11,3390	new state record	no		no	pos.	no	N.A.	positive	this study
8	EK3 WS	*Bufo bufo*	21.03.24	Germany	Bavaria, Altdorf, Winkelhaid	49,3937	11,3390	new state record	no		no	pos.	no	N.A.	positive	this study
9	EK4 WS	*Bufo bufo*	22.03.24	Germany	Bavaria, Altdorf, Winkelhaid	49,3937	11,3390	new state record	no		no	pos.	yes	PV005825	positive	this study
10	EK5 WS	*Bufo bufo*	23.03.24	Germany	Bavaria, Altdorf, Winkelhaid	49,3937	11,3390	new state record	no		no	pos.	no	N.A.	positive	this study
11	EK6 WS	*Bufo bufo*	24.03.24	Germany	Bavaria, Altdorf, Winkelhaid	49,3937	11,3390	new state record	no		no	pos.	no	N.A.	positive	this study
12	EK7 WS	*Bufo bufo*	25.03.24	Germany	Bavaria, Altdorf, Winkelhaid	49,3937	11,3390	new state record	no		no	pos.	no	N.A.	positive	this study
13	EK8 WS	*Bufo bufo*	26.03.24	Germany	Bavaria, Altdorf, Winkelhaid	49,3937	11,3390	new state record	no		no	pos.	no	N.A.	positive	this study
14	EK9 WS	*Bufo bufo*	27.03.24	Germany	Bavaria, Altdorf, Winkelhaid	49,3937	11,3390	new state record	no		no	pos.	no	N.A.	positive	this study
15	EK10 WS	*Bufo bufo*	28.03.24	Germany	Bavaria, Altdorf, Winkelhaid	49,3937	11,3390	new state record	no		no	pos.	no	N.A.	positive	this study
16	Irrel24.03.22	*Bufo bufo*	24.03.22	Germany	Rhineland-Palatinate, Irrel, Teufelsschlucht	49,8448	6,4357	new state record	yes		no	pos.	yes	PV005829	positive	this study
17	Igelsdorf	*Bufo bufo*	01.03.24	Germany	Bavaria, Igelsdorf	49,6460	11,0409	new state record	yes		no	neg.	no	N.A.	negative	this study
18	DB4EK1b	*Bufo bufo*	05.04.22	Germany	Rhineland-Palatinate, Densborn, Wackelbach	50,1227	6,5763	new state record	yes		no	neg.	no	N.A.	negative	this study
19	DB4EK2	*Bufo bufo*	05.04.22	Germany	Rhineland-Palatinate, Densborn, Wackelbach	50,1227	6,5763	new state record	yes		no	neg.	no	N.A.	negative	this study
20	DB2EK1	*Bufo bufo*	13.03.22	Germany	Rhineland-Palatinate, Densborn, Wackelbach	50,1227	6,5763	new state record	yes		no	pos.	no	N.A.	positive	this study
21	DB2EK3	*Bufo bufo*	13.03.22	Germany	Rhineland-Palatinate, Densborn, Wackelbach	50,1227	6,5763	new state record	yes		no	neg.	no	N.A.	negative	this study
22	DBEK1	*Bufo bufo*	13.03.22	Germany	Rhineland-Palatinate, Densborn, Wackelbach	50,1227	6,5763	new state record	yes		no	pos.	yes	PV005823	positive	this study
23	SP21c2a	*Bufo bufo*	22.08.21	Germany	Bavaria, Main-Spessart, Waldzell	49,9428	9,6137	new state record	no		no	neg.	no	N.A.	negative	this study
24	SPEK1290322	*Bufo bufo*	29.03.22	Germany	Rhineland-Palatinate, Densborn, Springelsbach	50,0413	6,4074	new state record	no		no	pos.	no	N.A.	positive	this study
25	SPEK2290322	*Bufo bufo*	29.03.22	Germany	Rhineland-Palatinate, Densborn, Springelsbach	50,1227	6,5763	new state record	no		no	pos.	yes	PV005827	positive	this study
26	Wi23EK1	*Bufo bufo*	01.03.23	Germany	Northrhine-Westphalia, NP Eifel, Winkelenberg	50,5969	6,4213	new state record	yes		no	neg.	no	N.A.	negative	this study
27	Wi23EK2	*Bufo bufo*	01.03.23	Germany	Northrhine-Westphalia, NP Eifel, Winkelenberg	50,5969	6,4213	new state record	yes		no	neg.	no	N.A.	negative	this study
28	Wi23EK3	*Bufo bufo*	01.03.23	Germany	Northrhine-Westphalia, NP Eifel, Winkelenberg	50,5969	6,4213	new state record	yes		no	pos.	yes	PV005824	positive	this study
29	WiEK1a21324	*Bufo bufo*	15.03.24	Germany	Northrhine-Westphalia, NP Eifel, Winkelenberg	50,5969	6,4213	new state record	yes		no	pos.	no	N.A.	positive	this study
30	WiEK1b21324	*Bufo bufo*	15.03.24	Germany	Northrhine-Westphalia, NP Eifel, Winkelenberg	50,5969	6,4213	new state record	yes		no	neg.	no	N.A.	negative	this study
31	TNPEK1a	*Bufo bufo*	13.04.22	Germany	Rhineland-Palatinate, Tongrube Niederprüm	50,1916	6,4065	new state record	no		no	neg.	no	N.A.	negative	this study
32	TNP22EK2a	*Bufo bufo*	13.04.22	Germany	Rhineland-Palatinate, Tongrube Niederprüm	50,1916	6,4065	new state record	no		no	neg.	no	N.A.	negative	this study
33	TNP22EK3a	*Bufo bufo*	13.04.22	Germany	Rhineland-Palatinate, Tongrube Niederprüm	50,1916	6,4065	new state record	no		no	neg.	no	N.A.	negative	this study
34	TNP23Ek1	*Bufo bufo*	21.04.23	Germany	Rhineland-Palatinate, Tongrube Niederprüm	50,1916	6,4065	new state record	no		no	pos.	no	N.A.	positive	this study
35	TNP23EK2	*Bufo bufo*	21.04.23	Germany	Rhineland-Palatinate, Tongrube Niederprüm	50,1916	6,4065	new state record	no		no	neg.	no	N.A.	negative	this study
36	TNP23EK3	*Bufo bufo*	21.04.23	Germany	Rhineland-Palatinate, Tongrube Niederprüm	50,1916	6,4065	new state record	no		no	pos.	yes	PV005826	positive	this study
37	TNP23EK4	*Bufo bufo*	07.05.23	Germany	Rhineland-Palatinate, Tongrube Niederprüm	50,1916	6,4065	new state record	no		no	neg.	no	N.A.	negative	this study
38	TNP23EK5	*Bufo bufo*	12.05.23	Germany	Rhineland-Palatinate, Tongrube Niederprüm	50,1916	6,4065	new state record	no		no	neg.	no	N.A.	negative	this study
39	TNP23EK6	*Bufo bufo*	12.05.23	Germany	Rhineland-Palatinate, Tongrube Niederprüm	50,1916	6,4065	new state record	no		no	neg.	no	N.A.	negative	this study
40	TNP23EK7	*Bufo bufo*	21.05.23	Germany	Rhineland-Palatinate, Tongrube Niederprüm	50,1916	6,4065	new state record	no		no	neg.	no	N.A.	negative	this study
41	TNP23EK8	*Bufo bufo*	21.05.23	Germany	Rhineland-Palatinate, Tongrube Niederprüm	50,1916	6,4065	new state record	no		no	neg.	no	N.A.	negative	this study
42	TNP23EK9	*Bufo bufo*	11.06.23	Germany	Rhineland-Palatinate, Tongrube Niederprüm	50,1916	6,4065	new state record	no		no	neg.	no	N.A.	negative	this study
43	TNP23EK10	*Bufo bufo*	19.06.23	Germany	Rhineland-Palatinate, Tongrube Niederprüm	50,1916	6,4065	new state record	no		no	neg.	no	N.A.	negative	this study
44	TNP23EK11	*Bufo bufo*	19.06.23	Germany	Rhineland-Palatinate, Tongrube Niederprüm	50,1916	6,4065	new state record	no		no	neg.	no	N.A.	negative	this study
45	TNP23EK12	*Bufo bufo*	21.06.23	Germany	Rhineland-Palatinate, Tongrube Niederprüm	50,1916	6,4065	new state record	no		no	neg.	no	N.A.	negative	this study
46	not applicable	*Bufo bufo*	05.03.24	Germany	Hesse, Idstein, Heftricher Moor	50,2072	8,3453	potential	yes	photo only	no	N.A.	no	N.A.	suspicious	this study
47	not applicable	*Bufo bufo*	2013	Germany	Bavaria, München, Aubinger Lohe	48,1724	11,4005	potential	yes	photo only	no	N.A.	no	N.A.	suspicious	this study
48	not applicable	*Bufo bufo*	06.04.22	Germany	Bavaria, Altdorf, Waldfriedhof	49,3937	11,3390	potential	yes	photo only	no	N.A.	no	N.A.	suspicious	this study
49	not applicable	*Bufo bufo*	11.04.22	Germany	Bavaria, Waldkraiburg, Stadtpark	48,2083	12,3953	potential	yes	photo only	no	N.A.	no	N.A.	suspicious	this study
50	not applicable	*Bufo bufo*	31.03.21	Luxembourg	Niederanven, Sennigerberg	49,6458	6,2205	new country record	yes	collection number: ZSM219/2024	yes	N.A.	no	N.A.	positive	this study
51	not applicable	*Bufo bufo*	17.03.24	Germany	Bavaria, Würzburg, Dürrbachtal	49,8195	9,9189	potential	yes	photo only	no	N.A.	no	N.A.	suspicious	this study
52	not applicable	*Bufo bufo*	20.03.23	Denmark	Haurvig, Hegnet	55,8425	8,1655	potential	yes		no	N.A.	no	N.A.	suspicious	https://www.gbif.org/occurrence/4067419543
53	not applicable	*Bufo bufo*	18.03.23	Germany	Braunschweig, Raffturm	52,2565	10,4534	potential	yes		no	N.A.	no	N.A.	suspicious	https://www.gbif.org/occurrence/4067157235
54	not applicable	*Bufo bufo*	13.03.23	United Kingdom	Shard End	52,4915	-1,7709	potential	yes		no	N.A.	no	N.A.	suspicious	https://www.gbif.org/occurrence/4062927274
55	not applicable	*Bufo bufo*	20.07.24	France	Xonrupt-Longemer, Chaume de Fachepremont	48,0472	6,9543	not applicable	yes	other skin disease	no	N.A.	no	N.A.	not applicable	https://www.gbif.org/occurrence/4911023544
56	not applicable	*Bufo bufo*	06.07.24	Slovenia	Ljubljana, Koseški boršt	46,0609	14,4686	not applicable	yes	other skin disease	no	N.A.	no	N.A.	not applicable	https://www.gbif.org/occurrence/4909295209
57	not applicable	*Bufo bufo*	18.05.24	United Kingdom	Grassington, Lower Hartlington	54,0444	-1,9450	not applicable	yes	other skin disease	no	N.A.	no	N.A.	not applicable	https://www.gbif.org/occurrence/4867853115
58	not applicable	*Bufo bufo*	11.05.24	United Kingdom	Aboyne, Upper Deeside and Donside, Derry Lodge	57,0066	-3,5449	not applicable	yes	other skin disease	no	N.A.	no	N.A.	not applicable	https://www.gbif.org/occurrence/4863342311
59	not applicable	*Bufo bufo*	01.05.24	Norway	Ullensaker, Krusestua	60,1588	11,1740	not applicable	yes	other skin disease	no	N.A.	no	N.A.	not applicable	https://www.gbif.org/occurrence/4855567705
60	not applicable	*Bufo bufo*	30.03.24	Lithuania	Gaurė, Pajkojai	55,1651	22,5106	potential	yes		no	N.A.	no	N.A.	suspicious	https://www.inatu…/photos/361591937
61	not applicable	*Bufo bufo*	30.03.24	Czechia	Mikulášovice, Salmov	50,9561	14,3893	potential	yes		no	N.A.	no	N.A.	suspicious	https://www.gbif.org/occurrence/4606828723
62	not applicable	*Bufo bufo*	20.03.24	United Kingdom	Thames, Appleton	51,7044	-1,3565	potential	yes		no	N.A.	no	N.A.	suspicious	https://www.gbif.org/occurrence/4600079085
63	not applicable	*Bufo bufo*	15.03.24	Germany	Rockhausen, Wasserburg Rockhausen	50,8978	11,0292	potential	yes		no	N.A.	no	N.A.	suspicious	https://www.gbif.org/occurrence/4599854745
64	not applicable	*Bufo bufo*	15.03.24	Czechia	Praha, Dufkovka	50,1042	14,3529	potential	yes		no	N.A.	no	N.A.	suspicious	https://www.inaturalist.org/photos/357744766
65	not applicable	*Bufo bufo*	15.03.24	Czechia	Praha, Dufkovka	50,1042	14,3529	potential	yes	BfHV uncertain	no	N.A.	no	N.A.	not applicable	https://www.inatu…/photos/357744794
66	not applicable	*Bufo bufo*	10.03.24	France	Val-Suzon, Sainte-Foy	47,4293	4,9406	potential	yes		no	N.A.	no	N.A.	suspicious	https://www.inatu…/photos/356486687
67	not applicable	*Bufo bufo*	07.03.24	Germany	Ulm, Lehr	48,4215	9,9643	potential	yes	BfHV uncertain	no	N.A.	no	N.A.	not applicable	https://www.inatu…/photos/356011381
68	not applicable	*Bufo bufo*	23.02.24	Austria	Wien, Rohdümpfelboden	48,1828	16,5414	not applicable	yes	other skin disease	no	N.A.	no	N.A.	not applicable	https://www.inatu…/photos/353278209
69	not applicable	*Bufo bufo*	14.08.23	Germany	Lenzkirch, Saig	47,8981	8,1779	not applicable	yes	other skin disease	no	N.A.	no	N.A.	not applicable	https://www.inatu…/photos/313667719
70	not applicable	*Bufo bufo*	11.07.23	Germany	Braunschweig, Dibbesdorf	52,3046	10,6116	not applicable	yes	other skin disease	no	N.A.	no	N.A.	not applicable	https://nabu-natu…n&bild=1553678962
71	not applicable	*Bufo bufo*	08.04.23	Lithuania	Rasos	54,6718	25,3266	potential	yes	BfHV uncertain	no	N.A.	no	N.A.	not applicable	https://www.inatu…/photos/265908541
72	not applicable	*Bufo bufo*	17.02.22	Germany	Walldorf	49,2924	8,6375	potential	yes	BfHV uncertain	no	N.A.	no	N.A.	not applicable	https://www.inatu…/photos/179754539
73	not applicable	*Bufo bufo*	15.12.23	The Netherlands	Voorst, Steenenkamer	52,2500	6,1500	not applicable	yes	other skin disease	no	N.A.	no	N.A.	not applicable	https://observati…otos/46503459.jpg
74	not applicable	*Bufo bufo*	01.03.22	United Kingdom	Raleigh, Otterton	50,6632	-3,3020	potential	yes	BfHV uncertain	no	N.A.	no	N.A.	not applicable	https://www.inaturalist.org/photos/181228338
75	not applicable	*Bufo bufo*	17.03.22	Germany	Freudenberg, Wolfskaute	50,8879	7,9488	potential	yes	BfHV uncertain	no	N.A.	no	N.A.	not applicable	https://www.inatu…/photos/183272004
76	not applicable	*Bufo bufo*	28.03.22	United Kingdom	Sutton, Piggford Moor	53,2372	-2,0526	potential	yes		no	N.A.	no	N.A.	suspicious	https://www.inatu…/photos/185037325
77	not applicable	*Bufo bufo*	24.03.22	Germany	Düsseldorf, Stockum	51,2566	6,7489	potential	yes	BfHV uncertain	no	N.A.	no	N.A.	not applicable	https://observati…otos/47045035.jpg
78	not applicable	*Bufo bufo*	18.03.22	Germany	Düsseldorf, Stockum	51,2565	6,7491	potential	yes		no	N.A.	no	N.A.	suspicious	https://observation.org/photos/47008754.jpg

79	not applicable	*Bufo bufo*	18.03.22	Germany	Düsseldorf, Stockum	51,2565	6,7491	potential	yes	BfHV uncertain	no	N.A.	no	N.A.	not applicable	https://observation.org/photos/47008752.jpg
80	not applicable	*Bufo bufo*	18.03.22	Germany	Düsseldorf, Stockum	51,2565	6,7491	potential	yes	2x BfHV	no	N.A.	no	N.A.	suspicious	https://observation.org/photos/47008751.jpg
81	not applicable	*Bufo bufo*	18.03.22	Germany	Düsseldorf, Golzheim	51,2498	6,7561	potential	yes		no	N.A.	no	N.A.	suspicious	https://observati…otos/47007011.jpg
82	not applicable	*Bufo bufo*	18.03.22	Germany	Düsseldorf, Stockum	51,2560	6,7496	potential	yes		no	N.A.	no	N.A.	suspicious	https://www.gbif.org/occurrence/3826102301
83	not applicable	*Bufo bufo*	23.05.24	United Kingdom	Gnosall & Woodseaves, Norbury	52,7995	-2,3044	not applicable	yes	other skin disease	no	N.A.	no	N.A.	not applicable	https://observati…otos/59075959.jpg
84	not applicable	*Bufo bufo*	15.12.23	The Netherlands	Venray, Endepoel	51,5500	5,9500	not applicable	yes	other skin disease	no	N.A.	no	N.A.	not applicable	https://observati…otos/57017196.jpg
85	not applicable	*Bufo bufo*	04.08.22	Sweden	Åmål, Ljusedal	58,8108	12,5928	not applicable	yes	other skin disease	no	N.A.	no	N.A.	not applicable	https://observati…otos/55888819.jpg
86	not applicable	*Bufo bufo*	08.09.22	Germany	Schwangau, Hohenschwangau	47,5592	10,7419	not applicable	yes	other skin disease	no	N.A.	no	N.A.	not applicable	https://www.inatu…/photos/224159839
87	not applicable	*Bufo bufo*	15.12.23	The Netherlands	Lingewaard, Bemmel	51,9000	5,9000	not applicable	yes	other skin disease	no	N.A.	no	N.A.	not applicable	https://observati…otos/55630469.jpg
88	not applicable	*Bufo bufo*	12.04.23	Sweden	Vimmerby, Örkullen	57,7322	15,7873	not applicable	yes	other skin disease	no	N.A.	no	N.A.	not applicable	https://observati…otos/55220310.jpg
89	not applicable	*Bufo bufo*	06.07.22	Slovakia	Skačany, Vrchy	48,6585	18,4003	not applicable	yes	other skin disease	no	N.A.	no	N.A.	not applicable	https://www.inatu…/photos/212011932
90	not applicable	*Bufo bufo*	15.12.23	Belgium	Hoogstraten, Wortel	51,4000	4,8000	not applicable	yes	other skin disease	no	N.A.	no	N.A.	not applicable	https://observati…otos/52574684.jpg
91	not applicable	*Bufo bufo*	23.05.22	Germany	Waldkirch	48,0883	7,9560	potential	yes	BfHV uncertain	no	N.A.	no	N.A.	not applicable	https://observati…otos/49869945.jpg
92	not applicable	*Bufo bufo*	15.12.23	The Netherlands	Zandvoort, Kruispan II	52,3500	4,5500	not applicable	yes	other skin disease	no	N.A.	no	N.A.	not applicable	https://observati…otos/42510612.jpg
93	not applicable	*Bufo bufo*	28.07.21	Germany	Fischbach bei Dahn, Unterpetersbächlerhof	49,0740	7,7075	not applicable	yes	other skin disease	no	N.A.	no	N.A.	not applicable	https://live.stat… 9d6c8d679a_b.jpg
94	not applicable	*Bufo bufo*	15.12.23	The Netherlands	Sint-Michielsgestel	51,6500	5,3500	potential	yes		no	N.A.	no	N.A.	suspicious	https://observati…otos/35574224.jpg
95	not applicable	*Bufo bufo*	15.12.23	The Netherlands	Sittard-Geleen, Graetheide	51,0000	5,8000	potential	yes		no	N.A.	no	N.A.	suspicious	https://observati…otos/36039065.jpg
96	not applicable	*Bufo bufo*	14.12.23	The Netherlands	Breda, Bolberg	51,5500	4,8500	not applicable	yes	other skin disease	no	N.A.	no	N.A.	not applicable	https://observati…otos/30216105.jpg
97	not applicable	*Bufo bufo*	14.12.23	The Netherlands	Wassenaar, Stevenshofjespolder	52,1500	4,4500	not applicable	yes	other skin disease	no	N.A.	no	N.A.	not applicable	https://observati…otos/29720900.jpg
98	not applicable	*Bufo bufo*	17.03.20	Germany	Remseck am Neckar, Rot	48,8824	9,2938	potential	yes		no	N.A.	no	N.A.	suspicious	https://live.stat… 622339e9db_b.jpg
99	not applicable	*Bufo bufo*	14.12.23	The Netherlands	Nederweert, Ospeldijk	51,3500	5,8000	not applicable	yes	other skin disease	no	N.A.	no	N.A.	not applicable	https://observati…otos/18607919.jpg
100	not applicable	*Bufo bufo*	09.09.18	Poland	Drawsko Pomorskie, Woliczno	53,4910	15,7183	not applicable	yes	other skin disease	no	N.A.	no	N.A.	not applicable	https://observati…otos/18260059.jpg
101	not applicable	*Bufo bufo*	21.06.18	Sweden	Åtvidaberg, Nedre Sundtorp	58,2313	15,8426	not applicable	yes	other skin disease	no	N.A.	no	N.A.	not applicable	https://www.inatu…g/photos/20175821
102	not applicable	*Bufo bufo*	14.12.23	The Netherlands	Bergen op Zoom, Spinolaberg	51,5000	4,2500	not applicable	yes	other skin disease	no	N.A.	no	N.A.	not applicable	https://observati…otos/11292363.jpg
103	not applicable	*Bufo bufo*	22.03.16	Germany	Langenthal	49,8211	7,5801	potential	yes		no	N.A.	no	N.A.	suspicious	https://nabu-natu…n&bild=1979544424
104	not applicable	*Bufo bufo*	26.03.16	Hungary	Miskolc, Miskolctapolca	48,0611	20,7482	potential	yes		no	N.A.	no	N.A.	suspicious	https://www.inatu…/photos/286941917
105	not applicable	*Bufo bufo*	26.03.24	Norway	Aremark, Nordre Størholt	59,3083	11,7561	potential	yes	other skin disease	no	N.A.	no	N.A.	not applicable	https://www.artso…59b82ab_image.jpg
106	not applicable	*Bufo bufo*	06.04.24	Germany	Weißenborn, Wochenendgebiet Eyerloch	50,9255	11,8844	potential	yes	BfHV uncertain	no	N.A.	no	N.A.	not applicable	https://observation.org/observation/303556411/
107	not applicable	*Bufo bufo*	21.03.24	The Netherlands	Wageningen, Oude Nude	51,9715	5,6448	potential	yes		no	N.A.	no	N.A.	suspicious	https://waarneming.nl/observation/301759376/
108	not applicable	*Bufo bufo*	21.03.24	Germany	Warstein, Im Kamp	51,4645	8,3956	potential	yes	BfHV uncertain	no	N.A.	no	N.A.	not applicable	https://observation.org/observation/301759441/
109	not applicable	*Bufo bufo*	21.03.24	Germany	Warstein, Im Kamp	51,4645	8,3966	potential	yes	BfHV uncertain	no	N.A.	no	N.A.	not applicable	https://observation.org/observation/301759472/
110	not applicable	*Bufo bufo*	19.03.24	Belgium	Neupré, Pied des Hayes de Mogen	50,5369	5,4419	potential	yes		no	N.A.	no	N.A.	suspicious	https://waarnemingen.be/observation/301790530/
111	not applicable	*Bufo bufo*	19.03.24	The Netherlands	Wageningen, Oude Nude	51,9750	5,6477	potential	yes		no	N.A.	no	N.A.	suspicious	https://waarneming.nl/observation/301559740/
112	not applicable	*Bufo bufo*	17.03.24	The Netherlands	Wageningen, Oude Nude	51,9679	5,6426	potential	yes		no	N.A.	no	N.A.	suspicious	https://waarneming.nl/observation/301227277/
113	not applicable	*Bufo bufo*	16.03.24	Germany	Bad Schandau, Vordere Partschenhörner	50,9106	14,2983	potential	yes		no	N.A.	no	N.A.	suspicious	https://observation.org/observation/301781369/
114	not applicable	*Bufo bufo*	16.03.24	Germany	Wernberg-Köblitz, Schmalhof	49,5327	12,1112	potential	yes		no	N.A.	no	N.A.	suspicious	https://observation.org/observation/301232206/
115	not applicable	*Bufo bufo*	16.03.24	Germany	Wernberg-Köblitz, Schmalhof	49,5333	12,1121	potential	yes		no	N.A.	no	N.A.	suspicious	https://observation.org/observation/301232205/
116	not applicable	*Bufo bufo*	13.03.24	Belgium	Wellen, Vrolingen	50,8460	5,3555	potential	yes		no	N.A.	no	N.A.	suspicious	https://waarnemingen.be/observation/300834209/
117	not applicable	*Bufo bufo*	11.03.24	The Netherlands	Tilburg, De Blaak	51,5486	5,0405	potential	yes	BfHV uncertain	no	N.A.	no	N.A.	not applicable	https://waarneming.nl/observation/300749224/
118	not applicable	*Bufo bufo*	09.03.24	Belgium	Damme, Den Hoorn	51,2339	3,3816	potential	yes	BfHV uncertain	no	N.A.	no	N.A.	not applicable	https://waarnemingen.be/observation/307930169/
119	not applicable	*Bufo bufo*	05.03.24	Germany	Seubersdorf i.d. OPf., Daßwang	49,1453	11,6577	potential	yes		no	N.A.	no	N.A.	suspicious	https://observation.org/observation/302250185/
120	not applicable	*Bufo bufo*	17.02.24	Belgium	Lochristi	51,0870	3,8354	potential	yes		no	N.A.	no	N.A.	suspicious	https://waarnemingen.be/observation/299131620/
121	not applicable	*Bufo bufo*	07.02.24	Belgium	Melle, Vogelhoek	51,0092	3,7758	potential	yes		no	N.A.	no	N.A.	suspicious	https://waarnemingen.be/observation/298385636/
122	not applicable	*Bufo bufo*	30.07.23	Germany	Berlin, Kaulsdorf	52,4963	13,5952	not applicable	yes	other skin disease	no	N.A.	no	N.A.	not applicable	https://observation.org/observation/282405217/
123	not applicable	*Bufo bufo*	22.07.23	Czechia	Bedřichov, Kristiánov	50,8233	15,1810	not applicable	yes	other skin disease	no	N.A.	no	N.A.	not applicable	https://observation.org/observation/281459026/
124	not applicable	*Bufo bufo*	19.07.23	Belgium	Lokeren, Naastveld	51,0831	4,0041	not applicable	yes	other skin disease	no	N.A.	no	N.A.	not applicable	https://waarnemingen.be/observation/281076414/
125	not applicable	*Bufo bufo*	13.07.23	Sweden	Ljusnarsberg, Myggsjögård	59,7908	14,9845	not applicable	yes	other skin disease	no	N.A.	no	N.A.	not applicable	https://observation.org/observation/280325774/
126	not applicable	*Bufo bufo*	01.07.23	N.A.	N.A.	N.A.	N.A.	not applicable	yes	other skin disease	no	N.A.	no	N.A.	not applicable	https://waarnemingen.be/observation/294441910/
127	not applicable	*Bufo bufo*	21.03.23	Belgium	Malle, Drieboomkensberg	51,2917	4,6546	potential	yes	BfHV uncertain	no	N.A.	no	N.A.	not applicable	https://waarnemingen.be/observation/265735355/
128	not applicable	*Bufo bufo*	20.03.23	Germany	Siegburg, Stallberg	50,8168	7,2290	potential	yes		no	N.A.	no	N.A.	suspicious	https://observation.org/observation/265624199/
129	not applicable	*Bufo bufo*	19.03.23	The Netherlands	Gooise Meren, Bussum	52,2679	5,1540	potential	yes		no	N.A.	no	N.A.	suspicious	https://waarneming.nl/observation/265616640/
130	not applicable	*Bufo bufo*	18.03.23	Belgium	Sainte-Ode, Amberloup	50,0286	5,5240	potential	yes		no	N.A.	no	N.A.	suspicious	https://waarnemingen.be/observation/265504134/
131	not applicable	*Bufo bufo*	17.03.23	The Netherlands	Almere, Almere Pampus	52,3671	5,1468	potential	yes		no	N.A.	no	N.A.	suspicious	https://waarneming.nl/observation/265408595/
132	not applicable	*Bufo bufo*	16.03.23	Germany	Düsseldorf, Stockum	51,2564	6,7491	potential	yes		no	N.A.	no	N.A.	suspicious	https://observation.org/observation/265298256/
133	not applicable	*Bufo bufo*	15.03.23	Belgium	Jabbeke, Zerkegem	51,1677	3,0819	potential	yes	BfHV uncertain	no	N.A.	no	N.A.	not applicable	https://waarnemingen.be/observation/265237119/
134	not applicable	*Bufo bufo*	23.02.23	Belgium	Gembloux, Grand-Manil	50,5489	4,6874	potential	yes	BfHV uncertain	no	N.A.	no	N.A.	not applicable	https://waarnemingen.be/observation/264162102/
135	not applicable	*Bufo bufo*	18.08.21	Belgium	Moerbeke	51,1733	3,9358	not applicable	yes	other skin disease	no	N.A.	no	N.A.	not applicable	https://waarnemingen.be/observation/223332676/
136	not applicable	*Bufo bufo*	15.08.21	The Netherlands	Bronckhorst, Noordink	52,0686	6,3207	not applicable	yes	other skin disease	no	N.A.	no	N.A.	not applicable	https://waarneming.nl/observation/223038013/
137	not applicable	*Bufo bufo*	09.08.21	The Netherlands	Nijmegen, Dukenburg	51,8051	5,7938	not applicable	yes	other skin disease	no	N.A.	no	N.A.	not applicable	https://waarneming.nl/observation/222512630/
138	not applicable	*Bufo bufo*	08.08.21	The Netherlands	Veere, Oostkapelle	51,5890	3,5697	not applicable	yes	other skin disease	no	N.A.	no	N.A.	not applicable	https://waarneming.nl/observation/222424292/
139	not applicable	*Bufo bufo*	30.07.21	The Netherlands	Deventer, Schalkhaar	52,2546	6,1989	not applicable	yes	other skin disease	no	N.A.	no	N.A.	not applicable	https://waarneming.nl/observation/221540326/
140	not applicable	*Bufo bufo*	28.07.21	The Netherlands	Baarn	52,2137	5,3053	not applicable	yes	other skin disease	no	N.A.	no	N.A.	not applicable	https://waarneming.nl/observation/221341528/
141	not applicable	*Bufo bufo*	24.07.21	Belgium	La Roche-en-Ardenne, Warempage	50,1189	5,6366	potential	yes		no	N.A.	no	N.A.	suspicious	https://waarnemingen.be/observation/237724180/
142	not applicable	*Bufo bufo*	23.07.21	Belgium	Sankt Vith#Saint-Vith, Neidingen	50,2498	6,1438	not applicable	yes	other skin disease	no	N.A.	no	N.A.	not applicable	https://waarnemingen.be/observation/220880804/
143	not applicable	*Bufo bufo*	04.07.21	The Netherlands	Westerveld, Westerzand	52,8128	6,2494	not applicable	yes	other skin disease	no	N.A.	no	N.A.	not applicable	https://waarneming.nl/observation/218988885/
144	not applicable	*Bufo bufo*	15.06.21	The Netherlands	Oosterhout, Seters	51,5963	4,8786	not applicable	yes	other skin disease	no	N.A.	no	N.A.	not applicable	https://waarneming.nl/observation/316159316/
145	not applicable	*Bufo bufo*	12.06.21	Belgium	Lennik, Gaasbeek	50,7974	4,1792	potential	yes	BfHV uncertain	no	N.A.	no	N.A.	not applicable	https://waarnemingen.be/observation/216742536/
146	not applicable	*Bufo bufo*	31.03.21	Belgium	Tellin, Resteigne	50,0862	5,1825	potential	yes		no	N.A.	no	N.A.	suspicious	https://waarnemingen.be/observation/209776808/
147	not applicable	*Bufo bufo*	24.03.21	Belgium	Gembloux, Grand-Manil	50,5496	4,6882	potential	yes		no	N.A.	no	N.A.	suspicious	https://waarnemingen.be/media/photo/33977931.jpg
148	not applicable	*Bufo bufo*	24.03.21	Belgium	Gembloux, Grand-Manil	50,5496	4,6882	potential	yes		no	N.A.	no	N.A.	suspicious	https://waarnemingen.be/media/photo/33977932.jpg
149	not applicable	*Bufo bufo*	24.03.21	Belgium	Gembloux, Grand-Manil	50,5483	4,6881	potential	yes		no	N.A.	no	N.A.	suspicious	https://waarnemingen.be/media/photo/33977811.jpg
150	not applicable	*Bufo bufo*	24.03.21	Belgium	Gembloux, Grand-Manil	50,5483	4,6881	potential	yes		no	N.A.	no	N.A.	suspicious	https://waarnemingen.be/media/photo/33977812.jpg
151	not applicable	*Bufo bufo*	23.03.21	Belgium	Gembloux, Grand-Manil	50,5494	4,6878	potential	yes	BfHV uncertain	no	N.A.	no	N.A.	not applicable	https://waarnemingen.be/media/photo/33947686.jpg
152	not applicable	*Bufo bufo*	23.03.21	Belgium	Gembloux, Grand-Manil	50,5494	4,6878	potential	yes		no	N.A.	no	N.A.	suspicious	https://waarnemingen.be/media/photo/33947687.jpg
153	not applicable	*Bufo bufo*	23.03.21	Belgium	Gembloux, Grand-Manil	50,5494	4,6878	potential	yes		no	N.A.	no	N.A.	suspicious	https://waarnemingen.be/media/photo/33947688.jpg
154	not applicable	*Bufo bufo*	23.03.21	Belgium	Gembloux, Grand-Manil	50,5494	4,6878	potential	yes	BfHV uncertain	no	N.A.	no	N.A.	not applicable	https://waarnemingen.be/media/photo/33947690.jpg
155	not applicable	*Bufo bufo*	04.03.21	N.A.	N.A.	N.A.	N.A.	not applicable	yes	other skin disease	no	N.A.	no	N.A.	not applicable	https://waarnemingen.be/observation/208244429/
156	not applicable	*Bufo bufo*	09.03.21	Belgium	Gembloux, Grand-Manil	50,5497	4,6879	potential	yes	BfHV uncertain	no	N.A.	no	N.A.	not applicable	https://waarnemingen.be/media/photo/33744143.jpg
157	not applicable	*Bufo bufo*	09.03.21	Belgium	Gembloux, Grand-Manil	50,5497	4,6879	potential	yes		no	N.A.	no	N.A.	suspicious	https://waarnemingen.be/media/photo/33744144.jpg
158	not applicable	*Bufo bufo*	23.03.21	Belgium	Gembloux, Grand-Manil	50,5492	4,6887	potential	yes	BfHV uncertain	no	N.A.	no	N.A.	not applicable	https://waarnemingen.be/media/photo/33947703.jpg
159	not applicable	*Bufo bufo*	23.03.21	Belgium	Gembloux, Grand-Manil	50,5492	4,6887	potential	yes		no	N.A.	no	N.A.	suspicious	https://waarnemingen.be/media/photo/33947704.jpg

160	not applicable	*Bufo bufo*	23.03.21	Belgium	Gembloux, Grand-Manil	50,5492	4,6887	potential	yes	BfHV uncertain	no	N.A.	no	N.A.	not applicable	https://waarnemingen.be/media/photo/33947705.jpg
161	not applicable	*Bufo bufo*	03.03.21	Belgium	Vilvoorde, Klein Hoogveld	50,9007	4,3922	potential	yes		no	N.A.	no	N.A.	suspicious	https://waarnemingen.be/observation/208253647/
162	not applicable	*Bufo bufo*	28.02.21	Belgium	Wommelgem, Laar	51,2145	4,4727	potential	yes		no	N.A.	no	N.A.	suspicious	https://waarnemingen.be/observation/208096570/
163	not applicable	*Bufo bufo*	24.02.21	Belgium	Gembloux, Grand-Manil	50,5500	4,6889	potential	yes		no	N.A.	no	N.A.	suspicious	https://waarnemingen.be/media/photo/33574704.jpg
164	not applicable	*Bufo bufo*	24.02.21	Belgium	Gembloux, Grand-Manil	50,5503	4,6889	potential	yes	BfHV uncertain	no	N.A.	no	N.A.	not applicable	https://waarnemingen.be/media/photo/33574658.jpg
165	not applicable	*Bufo bufo*	24.02.21	Belgium	Gembloux, Grand-Manil	50,5503	4,6889	potential	yes		no	N.A.	no	N.A.	suspicious	https://waarnemingen.be/media/photo/33574660.jpg
166	not applicable	*Bufo bufo*	24.02.21	Belgium	Vilvoorde, Klein Hoogveld	50,9008	4,3922	potential	yes		no	N.A.	no	N.A.	suspicious	https://waarnemingen.be/observation/207891659/
167	not applicable	*Bufo bufo*	24.02.21	Belgium	Herentals	51,1808	4,7998	potential	yes		no	N.A.	no	N.A.	suspicious	https://waarnemingen.be/observation/207838701/
168	not applicable	*Bufo bufo*	24.02.21	Belgium	Tongeren, Widooie	50,7799	5,4044	potential	yes		no	N.A.	no	N.A.	suspicious	https://waarnemingen.be/observation/207828020/
169	not applicable	*Bufo bufo*	23.02.21	Belgium	Tongeren, Widooie	50,7812	5,4055	potential	yes		no	N.A.	no	N.A.	suspicious	https://waarnemingen.be/observation/207769261/
170	not applicable	*Bufo bufo*	23.02.21	The Netherlands	Wageningen, Mobiel Ecodorp Ppauw	51,9649	5,6977	potential	yes		no	N.A.	no	N.A.	suspicious	https://waarneming.nl/observation/207769301/
171	not applicable	*Bufo bufo*	21.02.21	Belgium	Tongeren, Neerrepen	50,8110	5,4503	potential	yes	BfHV uncertain	no	N.A.	no	N.A.	not applicable	https://waarnemingen.be/observation/207704932/
172	not applicable	*Bufo bufo*	04.08.22	Sweden	Åmål, Ljusedal	58,8108	12,5928	not applicable	yes	other skin disease	no	N.A.	no	N.A.	not applicable	https://observation.org/observation/251324833/
173	not applicable	*Bufo bufo*	04.08.22	Belgium	Malmedy, Bévercé	50,4383	6,0433	not applicable	yes	other skin disease	no	N.A.	no	N.A.	not applicable	https://waarnemingen.be/observation/251332824/
174	not applicable	*Bufo bufo*	26.07.22	The Netherlands	Eindhoven, Stratum	51,4078	5,5041	not applicable	yes	other skin disease	no	N.A.	no	N.A.	not applicable	https://waarneming.nl/observation/251803822/
175	not applicable	*Bufo bufo*	25.07.22	Sweden	Vimmerby, Örkullen	57,7322	15,7873	not applicable	yes	other skin disease	no	N.A.	no	N.A.	not applicable	https://observation.org/observation/250316645/
176	not applicable	*Bufo bufo*	11.07.22	France	Wallers, Arenberg	50,3978	3,4143	not applicable	yes	other skin disease	no	N.A.	no	N.A.	not applicable	https://observation.org/observation/248679258/
177	not applicable	*Bufo bufo*	23.06.22	The Netherlands	Oldenzaal, Recreatiepark Het Hulsbeek	52,3042	6,8756	not applicable	yes	other skin disease	no	N.A.	no	N.A.	not applicable	https://waarneming.nl/observation/246565370/
178	not applicable	*Bufo bufo*	13.04.22	The Netherlands	Raalte, Schoonheterheide	52,3629	6,3366	potential	yes		no	N.A.	no	N.A.	suspicious	https://waarneming.nl/observation/238290563/
179	not applicable	*Bufo bufo*	06.04.22	Belgium	Ieper, Sint-Elooi	50,8083	2,9139	potential	yes		no	N.A.	no	N.A.	suspicious	https://waarnemingen.be/observation/236928398/
180	not applicable	*Bufo bufo*	29.03.22	Austria	Linz, Kleinmünchen	48,2555	14,3049	potential	yes		no	N.A.	no	N.A.	suspicious	https://observation.org/observation/236586716/
181	not applicable	*Bufo bufo*	24.03.22	The Netherlands	Dantumadiel, Readtsjerk	53,2647	5,9514	potential	yes		no	N.A.	no	N.A.	suspicious	https://waarneming.nl/observation/236181288/
182	not applicable	*Bufo bufo*	22.03.22	Belgium	Lint	51,1321	4,5202	potential	yes		no	N.A.	no	N.A.	suspicious	https://waarnemingen.be/observation/235919416/
183	not applicable	*Bufo bufo*	13.03.22	Belgium	Quiévrain, Audregnies	50,3778	3,7236	potential	yes	4x BfHV	no	N.A.	no	N.A.	suspicious	https://waarnemingen.be/observation/235317198/
184	not applicable	*Bufo bufo*	13.03.22	Belgium	Quiévrain, Audregnies	50,3781	3,7240	potential	yes		no	N.A.	no	N.A.	suspicious	https://waarnemingen.be/observation/235317158/
185	not applicable	*Bufo bufo*	13.03.22	Belgium	Quiévrain, Audregnies	50,3785	3,7261	potential	yes	2x BfHV	no	N.A.	no	N.A.	suspicious	https://waarnemingen.be/observation/235315633/
186	not applicable	*Bufo bufo*	13.03.22	Belgium	Honnelles, Montignies-sur-Roc	50,3761	3,7288	potential	yes		no	N.A.	no	N.A.	suspicious	https://waarnemingen.be/observation/235312016/
187	not applicable	*Bufo bufo*	13.03.22	Belgium	Honnelles, Montignies-sur-Roc	50,3766	3,7288	potential	yes		no	N.A.	no	N.A.	suspicious	https://waarnemingen.be/observation/235311801/
188	not applicable	*Bufo bufo*	13.03.22	Belgium	Quiévrain, Audregnies	50,3778	3,7281	potential	yes	1x BfHV	no	N.A.	no	N.A.	suspicious	https://waarnemingen.be/observation/235310710/
189	not applicable	*Bufo bufo*	12.03.22	Belgium	Ath, Coucou	50,6675	3,6945	potential	yes		no	N.A.	no	N.A.	suspicious	https://waarnemingen.be/observation/235165335/
190	not applicable	*Bufo bufo*	22.02.22	Belgium	Boom, Bosstraat	51,0879	4,3988	potential	yes		no	N.A.	no	N.A.	suspicious	https://waarnemingen.be/observation/234147783/
191	not applicable	*Bufo bufo*	22.02.22	Belgium	Boom, Bosstraat	51,0876	4,3990	potential	yes		no	N.A.	no	N.A.	suspicious	https://waarnemingen.be/observation/234147708/
192	not applicable	*Bufo bufo*	22.02.22	Belgium	Boom, Bosstraat	51,0879	4,3989	potential	yes		no	N.A.	no	N.A.	suspicious	https://waarnemingen.be/observation/234147770/
193	not applicable	*Bufo bufo*	09.02.22	The Netherlands	Deventer, Schalkhaar	52,2786	6,1865	potential	yes		no	N.A.	no	N.A.	suspicious	https://waarneming.nl/observation/233576167/
194	not applicable	*Bufo bufo*	10.09.21	Belgium	Beloeil, Les Écacheries	50,5257	3,7447	potential	yes		no	N.A.	no	N.A.	suspicious	https://waarnemingen.be/observation/225533542/
195	not applicable	*Bufo bufo*	10.09.21	The Netherlands	Venlo, Leemhorst	51,3169	6,1426	potential	yes		no	N.A.	no	N.A.	suspicious	https://waarneming.nl/observation/225336644/
196	not applicable	*Bufo bufo*	25.02.19	Belgium	Wommelgem, Laar	51,2158	4,4731	potential	yes		no	N.A.	no	N.A.	suspicious	https://waarnemingen.be/observation/168001614/
197	not applicable	*Bufo bufo*	02.03.19	Belgium	Olen, Onze-Lieve-Vrouw-Olen	51,1668	4,8941	potential	yes		no	N.A.	no	N.A.	suspicious	https://waarnemingen.be/observation/168237698/
198	not applicable	*Bufo bufo*	03.03.19	Belgium	Kaprijke, Lembeke	51,1796	3,6184	potential	yes		no	N.A.	no	N.A.	suspicious	https://waarnemingen.be/observation/168259107/
199	not applicable	*Bufo bufo*	30.07.19	Sweden	Vansbro, Sälsriset	60,3742	14,4029	not applicable	yes	other skin disease	no	N.A.	no	N.A.	not applicable	https://observation.org/observation/230115210/
200	not applicable	*Bufo bufo*	16.02.20	Belgium	Edegem	51,1472	4,4564	potential	yes		no	N.A.	no	N.A.	suspicious	https://waarnemingen.be/observation/185573522/
201	not applicable	*Bufo bufo*	24.02.20	Belgium	Knesselare, Berhoutbos	51,1338	3,5077	potential	yes		no	N.A.	no	N.A.	suspicious	https://waarnemingen.be/observation/185805974/
202	not applicable	*Bufo bufo*	04.03.20	Belgium	Wommelgem, Laar	51,2157	4,4767	potential	yes		no	N.A.	no	N.A.	suspicious	https://waarnemingen.be/observation/186147718/
203	not applicable	*Bufo bufo*	04.03.20	Belgium	Wommelgem, Laar	51,2117	4,4707	potential	yes		no	N.A.	no	N.A.	suspicious	https://waarnemingen.be/observation/186147741/
204	not applicable	*Bufo bufo*	10.03.20	Belgium	Tielt, Schuiferskapelle	51,0397	3,3468	potential	yes		no	N.A.	no	N.A.	suspicious	https://waarnemingen.be/observation/186386087/
205	not applicable	*Bufo bufo*	11.03.20	Belgium	Damme	51,2468	3,2794	potential	yes		no	N.A.	no	N.A.	suspicious	https://waarnemingen.be/observation/186424212/
206	not applicable	*Bufo bufo*	11.03.20	Belgium	Kortemark, Bescheewege	51,0404	2,9929	potential	yes		no	N.A.	no	N.A.	suspicious	https://waarnemingen.be/observation/186429805/
207	not applicable	*Bufo bufo*	11.03.20	Belgium	Lille, Wechelderzande	51,2705	4,8076	potential	yes		no	N.A.	no	N.A.	suspicious	https://waarnemingen.be/observation/186428201/
208	not applicable	*Bufo bufo*	17.03.20	Germany	Stolberg (Rhld.), Städteregion Aachen	50,7618	6,1942	potential	yes		no	N.A.	no	N.A.	suspicious	https://observation.org/observation/194717061/
209	not applicable	*Bufo bufo*	18.06.20	The Netherlands	Oude IJsselstreek, Etten	51,9241	6,3333	not applicable	yes	other skin disease	no	N.A.	no	N.A.	not applicable	https://waarneming.nl/observation/194367504/
210	not applicable	*Bufo bufo*	30.06.20	The Netherlands	Aa en Hunze, Gasteren	53,0424	6,6581	not applicable	yes	other skin disease	no	N.A.	no	N.A.	not applicable	https://waarneming.nl/observation/195829358/
211	not applicable	*Bufo bufo*	17.07.20	The Netherlands	Westvoorne, Rockanje	51,8469	4,0779	not applicable	yes	other skin disease	no	N.A.	no	N.A.	not applicable	https://waarneming.nl/observation/196586968/
212	not applicable	*Bufo bufo*	25.08.20	Belgium	Maasmechelen, Meisberg	51,0027	5,6734	not applicable	yes	other skin disease	no	N.A.	no	N.A.	not applicable	https://waarnemingen.be/observation/199062606/
213	not applicable	*Bufo bufo*	01.09.20	The Netherlands	Steenwijkerland, Oldemarkt	52,8242	5,9746	not applicable	yes	other skin disease	no	N.A.	no	N.A.	not applicable	https://waarneming.nl/observation/199400366/
214	not applicable	*Bufo bufo*	29.06.14	The Netherlands	Tilburg, Vossenberg	51,6093	5,0299	not applicable	yes	other skin disease	no	N.A.	no	N.A.	not applicable	https://waarneming.nl/observation/85911871/
215	not applicable	*Bufo bufo*	10.03.15	Belgium	Berlare, Overmere	51,0446	3,9500	potential	yes		no	N.A.	no	N.A.	suspicious	https://waarnemingen.be/observation/98612117/
216	not applicable	*Bufo bufo*	17.03.15	The Netherlands	Woerden	52,0745	4,8637	potential	yes	BfHV uncertain	no	N.A.	no	N.A.	not applicable	https://waarneming.nl/observation/100107232/
217	not applicable	*Bufo bufo*	08.04.15	The Netherlands	Borsele, Heinkenszand	51,4673	3,8201	potential	yes		no	N.A.	no	N.A.	suspicious	https://waarneming.nl/observation/100691440/
218	not applicable	*Bufo bufo*	10.04.15	Belgium	Tielt-Winge, Tielt	50,9332	4,8860	potential	yes	BfHV uncertain	no	N.A.	no	N.A.	not applicable	https://waarnemingen.be/media/photo/8255815.jpg
219	not applicable	*Bufo bufo*	10.04.15	Belgium	Tielt-Winge, Tielt	50,9332	4,8860	potential	yes		no	N.A.	no	N.A.	suspicious	https://waarnemingen.be/media/photo/8255816.jpg
220	not applicable	*Bufo bufo*	10.04.15	Belgium	Tielt-Winge, Tielt	50,9332	4,8860	potential	yes		no	N.A.	no	N.A.	suspicious	https://waarnemingen.be/media/photo/8255817.jpg
221	not applicable	*Bufo bufo*	12.04.15	The Netherlands	Vaals, Camerig	50,7682	5,9293	potential	yes	2x BfHV	no	N.A.	no	N.A.	suspicious	https://waarneming.nl/observation/100826421/
222	not applicable	*Bufo bufo*	02.04.16	The Netherlands	Nederweert, Hulsen	51,2820	5,7658	potential	yes		no	N.A.	no	N.A.	suspicious	https://waarneming.nl/observation/116012267/
223	not applicable	*Bufo bufo*	08.03.17	Belgium	Vilvoorde, Klein Hoogveld	50,9037	4,3888	potential	yes		no	N.A.	no	N.A.	suspicious	https://waarnemingen.be/media/photo/12934617.jpg
224	not applicable	*Bufo bufo*	08.03.17	Belgium	Vilvoorde, Klein Hoogveld	50,9037	4,3888	potential	yes		no	N.A.	no	N.A.	suspicious	https://waarnemingen.be/media/photo/12934620.jpg
225	not applicable	*Bufo bufo*	08.03.17	Belgium	Vilvoorde, Klein Hoogveld	50,9037	4,3888	potential	yes	BfHV uncertain	no	N.A.	no	N.A.	not applicable	https://waarnemingen.be/media/photo/12934623.jpg
226	not applicable	*Bufo bufo*	08.03.17	Belgium	Vilvoorde, Klein Hoogveld	50,9037	4,3888	potential	yes	BfHV uncertain	no	N.A.	no	N.A.	not applicable	https://waarnemingen.be/media/photo/12934607.jpg
227	not applicable	*Bufo bufo*	08.03.17	Belgium	Vilvoorde, Klein Hoogveld	50,9037	4,3888	potential	yes	1x BfHV	no	N.A.	no	N.A.	suspicious	https://waarnemingen.be/media/photo/12934611.jpg
228	not applicable	*Bufo bufo*	08.03.17	Belgium	Vilvoorde, Klein Hoogveld	50,9037	4,3888	potential	yes	BfHV uncertain	no	N.A.	no	N.A.	not applicable	https://waarnemingen.be/media/photo/12934612.jpg
229	not applicable	*Bufo bufo*	18.03.17	Belgium	Gavere, Baaigem	50,9490	3,7201	potential	yes	2x BfHV	no	N.A.	no	N.A.	suspicious	https://waarnemingen.be/observation/135569982/
230	not applicable	*Bufo bufo*	10.07.17	The Netherlands	Grave, Zaalheuvel	51,7447	5,7148	potential	yes	BfHV uncertain, other skin disease	no	N.A.	no	N.A.	not applicable	https://waarneming.nl/observation/141442631/
231	not applicable	*Bufo bufo*	07.04.09	The Netherlands	Haaren, Zandkant	51,6294	5,1650	potential	yes		no	N.A.	no	N.A.	suspicious	https://waarneming.nl/observation/42405689/
232	not applicable	*Bufo bufo*	15.03.07	The Netherlands	Grave, Nieuw-Gassel	51,7226	5,7662	potential	yes		no	N.A.	no	N.A.	suspicious	https://waarneming.nl/observation/2150145/
233	not applicable	*Bufo bufo*	17.08.06	Germany	Asselfingen, Speckplätze	48,4830	10,2316	potential	yes	other skin disease	no	N.A.	no	N.A.	not applicable	https://observation.org/observation/324466142/
234	not applicable	*Bufo bufo*	01.04.14	The Netherlands	Wageningen	51,9627	5,6815	potential	yes	1x BfHV	no	N.A.	no	N.A.	suspicious	https://waarneming.nl/observation/82931406/
235	not applicable	*Bufo bufo*	29.03.14	The Netherlands	Lingewaal, Spijk	51,8486	5,0263	potential	yes	BfHV uncertain	no	N.A.	no	N.A.	not applicable	https://waarneming.nl/observation/82812135/
236	not applicable	*Bufo bufo*	18.03.14	Belgium	Peer	51,1322	5,4364	potential	yes		no	N.A.	no	N.A.	suspicious	https://waarnemingen.be/media/photo/6211207.jpg
237	not applicable	*Bufo bufo*	18.03.14	Belgium	Peer	51,1322	5,4364	potential	yes		no	N.A.	no	N.A.	suspicious	https://waarnemingen.be/media/photo/6211208.jpg
238	not applicable	*Bufo bufo*	18.03.14	Belgium	Peer	51,1322	5,4364	potential	yes		no	N.A.	no	N.A.	suspicious	https://waarnemingen.be/media/photo/6211210.jpg
239	not applicable	*Bufo bufo*	18.03.14	Belgium	Lierde, Sint-Maria-Lierde	50,8310	3,8343	potential	yes	BfHV uncertain, other skin disease	no	N.A.	no	N.A.	not applicable	https://waarnemingen.be/observation/82522131/
240	not applicable	*Bufo bufo*	14.04.13	The Netherlands	Bloemendaal, Overveen	52,3814	4,6203	potential	yes		no	N.A.	no	N.A.	suspicious	https://waarneming.nl/observation/75117581/

241	not applicable	*Bufo bufo*	13.04.13	Belgium	Brugelette, Attre	50,6131	3,8357	potential	yes	BfHV uncertain	no	N.A.	no	N.A.	not applicable	https://waarnemingen.be/observation/75042252/
242	not applicable	*Bufo bufo*	06.03.13	Belgium	Heist-op-den-Berg, Werft	51,0855	4,7031	potential	yes	2x BfHV	no	N.A.	no	N.A.	suspicious	https://waarnemingen.be/media/photo/4450187.jpg
243	not applicable	*Bufo bufo*	06.03.13	Belgium	Heist-op-den-Berg, Werft	51,0855	4,7031	potential	yes		no	N.A.	no	N.A.	suspicious	https://waarnemingen.be/media/photo/4450191.jpg
244	not applicable	*Bufo bufo*	06.03.13	Belgium	Heist-op-den-Berg, Werft	51,0855	4,7031	potential	yes		no	N.A.	no	N.A.	suspicious	https://waarnemingen.be/media/photo/4450195.jpg
245	not applicable	*Bufo bufo*	16.03.12	Belgium	Antwerpen, Edison	51,2788	4,4020	potential	yes		no	N.A.	no	N.A.	suspicious	https://waarnemingen.be/observation/63725341/
246	not applicable	*Bufo bufo*	10.03.12	The Netherlands	Bergen, Egmond-Binnen	52,5937	4,6366	potential	yes		no	N.A.	no	N.A.	suspicious	https://waarneming.nl/observation/63612957/
247	not applicable	*Bufo bufo*	03.03.12	Belgium	Ganshoren	50,8811	4,3120	potential	yes		no	N.A.	no	N.A.	suspicious	https://waarnemingen.be/observation/63465246/
248	not applicable	*Bufo bufo*	29.02.12	The Netherlands	Enkhuizen	52,7124	5,2843	potential	yes	BfHV uncertain	no	N.A.	no	N.A.	not applicable	https://waarneming.nl/observation/63407480/
249	not applicable	*Bufo bufo*	01.04.11	Germany	Altena, Hardt	51,3038	7,7123	potential	yes	1x BfHV	no	N.A.	no	N.A.	suspicious	https://observation.org/observation/259423200/
250	not applicable	*Bufo bufo*	31.03.18	The Netherlands	Venlo, De Drie Dennen	51,4239	6,2081	potential	yes	2x BfHV	no	N.A.	no	N.A.	suspicious	https://waarneming.nl/observation/150147653/
251	not applicable	*Bufo bufo*	30.03.18	Germany	Coesfeld, Letter Bruch	51,8979	7,1256	potential	yes	1x BfHV	no	N.A.	no	N.A.	suspicious	https://observation.org/observation/150115054/
252	not applicable	*Bufo bufo*	10.03.18	The Netherlands	Boxmeer, Hoogeind	51,7026	5,9522	potential	yes		no	N.A.	no	N.A.	suspicious	https://waarneming.nl/media/photo/16082405.jpg
253	not applicable	*Bufo bufo*	10.03.18	Belgium	Knesselare, Berhoutbos	51,1338	3,5063	potential	yes	1x BfHV	no	N.A.	no	N.A.	suspicious	https://waarnemingen.be/observation/149375671/
254	not applicable	*Bufo bufo*	30.07.24	Belgium	Dessel, Brasel	51,2301	5,0449	not applicable	yes	other skin disease	no	N.A.	no	N.A.	not applicable	https://waarnemingen.be/observation/322030762/
255	not applicable	*Bufo bufo*	05.08.24	Sweden	Värmdö, Löknäs	59,3734	18,6593	not applicable	yes	other skin disease	no	N.A.	no	N.A.	not applicable	https://observation.org/observation/322830211/
256	not applicable	*Bufo bufo*	09.08.24	The Netherlands	Westerveld, Wateren	52,9340	6,2906	not applicable	yes	other skin disease	no	N.A.	no	N.A.	not applicable	https://waarneming.nl/observation/323572489/
257	not applicable	*Bufo bufo*	11.08.24	Austria	Altaussee, Sechserfleck	47,6572	13,7767	not applicable	yes	other skin disease	no	N.A.	no	N.A.	not applicable	https://observation.org/observation/323642659/
258	not applicable	*Bufo bufo*	12.08.24	The Netherlands	Venlo, Santfort	51,3948	6,1231	not applicable	yes	other skin disease	no	N.A.	no	N.A.	not applicable	https://waarneming.nl/observation/323854591/
259	not applicable	*Bufo bufo*	17.08.24	The Netherlands	Deurne, De Voorstad	51,4187	5,8600	not applicable	yes	other skin disease	no	N.A.	no	N.A.	not applicable	https://waarneming.nl/observation/324758320/
260	not applicable	*Bufo bufo*	17.08.24	Germany	Schliersee, Spitzingsee	47,6637	11,8809	not applicable	yes	other skin disease	no	N.A.	no	N.A.	not applicable	https://observation.org/observation/324974175/
261	not applicable	*Bufo bufo*	20.08.24	The Netherlands	Wassenaar, Meijendel	52,1267	4,3263	not applicable	yes	other skin disease	no	N.A.	no	N.A.	not applicable	https://waarneming.nl/observation/325161916/
262	not applicable	*Bufo bufo*	20.08.24	The Netherlands	Emmen, Meerstalblok	52,6990	7,0608	not applicable	yes	other skin disease	no	N.A.	no	N.A.	not applicable	https://waarneming.nl/observation/325180981/
263	not applicable	*Bufo bufo*	24.08.24	Germany	Lissendorf	50,3179	6,6089	potential	yes		no	N.A.	no	N.A.	suspicious	https://observation.org/observation/325570715/
264	not applicable	*Bufo bufo*	23.06.24	Germany	Kirchenpingarten, Fuchsendorf	49,9158	11,8317	not applicable	yes	other skin disease	no	N.A.	no	N.A.	not applicable	https://observation.org/observation/315694606/
265	not applicable	*Bufo bufo*	27.06.24	The Netherlands	Bronckhorst, Oosterwijk	52,0319	6,3513	not applicable	yes	other skin disease	no	N.A.	no	N.A.	not applicable	https://waarneming.nl/observation/316496339/
266	not applicable	*Bufo bufo*	02.07.24	The Netherlands	Westland, 's-Gravenzande	52,0087	4,1711	not applicable	yes	other skin disease	no	N.A.	no	N.A.	not applicable	https://waarneming.nl/observation/317179668/
267	not applicable	*Bufo bufo*	03.07.24	The Netherlands	Bloemendaal, Grote Vlak	52,4189	4,6098	not applicable	yes	other skin disease	no	N.A.	no	N.A.	not applicable	https://waarneming.nl/media/photo/95992877.jpg
268	not applicable	*Bufo bufo*	12.07.24	Germany	Moringen, An der Landwehr	51,7391	9,8097	not applicable	yes	other skin disease	no	N.A.	no	N.A.	not applicable	https://observation.org/observation/323825457/
269	not applicable	*Bufo bufo*	14.07.24	Germany	Nettetal, Schnick	51,3535	6,2369	potential	yes	BfHV uncertain	no	N.A.	no	N.A.	not applicable	https://observation.org/observation/318967509/
270	not applicable	*Bufo bufo*	19.07.24	Belgium	Olen, Sint-Jozef-Olen	51,2080	4,8818	not applicable	yes	other skin disease	no	N.A.	no	N.A.	not applicable	https://waarnemingen.be/observation/319888486/
271	not applicable	*Bufo bufo*	21.07.24	Belgium	Bilzen, Munsterbilzen	50,9015	5,5457	not applicable	yes	other skin disease	no	N.A.	no	N.A.	not applicable	https://waarnemingen.be/observation/320254544/
272	not applicable	*Bufo bufo*	21.07.24	Belgium	Maasmechelen, Meisberg	50,9703	5,6467	not applicable	yes	other skin disease	no	N.A.	no	N.A.	not applicable	https://waarnemingen.be/observation/320292773/
273	not applicable	*Bufotes v. sitibundi*	18.05.24	Georgia	Krtsanisi, Ponichala Reserve	41,6317	44,9305	potential	yes	BfHV1 uncertain	no	N.A.	no	N.A.	not applicable	https://www.gbif.org/occurrence/4867959191
274	not applicable	*Bufotes v. balearicu*	01.07.24	Italy	Bergamo	45,7058	9,6385	potential	yes	BfHV1 uncertain	no	N.A.	no	N.A.	not applicable	https://www.gbif.org/occurrence/4904099625
275	not applicable	*Bufotes v. sitibundi*	26.05.23	Russia	Buynakskiy rayon	43,0642	47,2296	not applicable	yes	other skin disease	no	N.A.	no	N.A.	not applicable	https://www.gbif.org/occurrence/4137807218
276	not applicable	*Bufotes viridis*	19.06.23	Türkiye	Burdur	37,3682	29,9888	not applicable	yes	other skin disease	no	N.A.	no	N.A.	not applicable	https://www.gbif.org/occurrence/4138445009
277	not applicable	*Bufotes viridis*	23.07.22	Germany	Bayern	48,3157	11,6627	not applicable	yes	other skin disease	no	N.A.	no	N.A.	not applicable	https://www.gbif.org/occurrence/3873077261
278	not applicable	*Bufotes viridis*	25.09.21	Poland	Warsaw West County	52,2370	20,7800	not applicable	yes	other skin disease	no	N.A.	no	N.A.	not applicable	https://www.gbif.org/occurrence/3384233168
279	not applicable	*Bufotes v. sitibundi*	03.05.19	Iran	Tafresh	34,7324	4,9873	not applicable	yes	other skin disease	no	N.A.	no	N.A.	not applicable	https://www.gbif.org/occurrence/2619946406
280	not applicable	*Bufotes v. balearicu*	29.06.15	Italy	Milano	45,4894	8,8857	not applicable	yes	other skin disease	no	N.A.	no	N.A.	not applicable	https://www.gbif.org/occurrence/4420697818
281	not applicable	*Bufotes viridis*	28.06.12	Italy	Provincia di Pordenone	46,0620	12,7982	not applicable	yes	other skin disease	no	N.A.	no	N.A.	not applicable	https://www.gbif.org/occurrence/1562941152
282	not applicable	*Bufotes viridis*	01.07.23	Italy	Como	45,8024	9,0817	not applicable	yes	other skin disease	no	N.A.	no	N.A.	not applicable	https://observation.org/observation/278781642/
283	not applicable	*Bufotes viridis*	10.07.23	Germany	Bonn, Volmershoven	50,6817	7,0147	not applicable	yes	other skin disease	no	N.A.	no	N.A.	not applicable	https://observation.org/observation/279877714/
284	not applicable	*Bufotes viridis*	26.04.22	Georgia	Almasiani, Kazbegi	42,5734	44,4747	potential	yes	BfHV1 uncertain	no	N.A.	no	N.A.	not applicable	https://observation.org/observation/239149094/
285	not applicable	*Bufo bufo*	18.03.24	Germany	Baden-Württemberg, Lonsee-Urspring	48,5503	9,8921	potential	yes		no	N.A.	no	N.A.	suspicious	this study
286	not applicable	*Bufo bufo*	2012	Germany	Northrhine-Westphalia, Kevelaer	51,5919	6,2557	potential	yes		no	N.A.	no	N.A.	suspicious	this study
287	not applicable	*Bufo bufo*	29.03.22	Germany	Karlsbad-Ittersbach, Rhododendronpark	48,4834	10,5581	potential	yes		no	N.A.	no	N.A.	suspicious	this study
288	not applicable	*Bufo bufo*	13.03.24	Germany	Solingen, Theegarten	51,1753	7,1113	potential	yes		no	N.A.	no	N.A.	suspicious	this study
289	not applicable	*Bufo bufo*	17.03.24	Germany	Hesse, Steinbrücker Teich	49,8912	8,6985	potential	yes		no	N.A.	no	N.A.	suspicious	this study
290	not applicable	*Bufo bufo*	19.03.24	Germany	Hesse, Steinbrücker Teich	49,8912	8,6985	potential	yes		no	N.A.	no	N.A.	suspicious	this study
291	not applicable	*Bufo bufo*	28.03.18	Germany	Kaolingrube Ortenberg	50,3568	9,6240	first german record	yes		no	pos.	yes	MT975973	positive	Eisenberg et al. 2018
292	not applicable	*Bufo bufo*	14.03.07	The Netherlands	Herpeduin, Schaijk	51,7531	5,5872	potential	yes		no	N.A.	no	N.A.	suspicious	this study
293	not applicable	*Epidalea calamita*	11.05.24	Spain	Communidad de Madrird, Madrid	40,8346	-3,9596	not applicable		other skin disease	no	N.A.	no	N.A.	not applicable	https://www.gbif.org/occurrence/4863860880
294	not applicable	*Epidalea calamita*	13.05.24	France	Languedoc-Roussillon, Pujaut	43,9857	4,7289	not applicable		other skin disease	no	N.A.	no	N.A.	not applicable	https://www.gbif.org/occurrence/4867769167
295	not applicable	*Epidalea calamita*	19.05.24	France	Provence-Alpes-Côte d'Azur, Roquebrune-sur-Argens	43,4683	6,6229	not applicable		other skin disease	no	N.A.	no	N.A.	not applicable	https://www.gbif.org/occurrence/4872074336
296	not applicable	*Epidalea calamita*	14.07.24	The Netherlands	Gelderland, Millingen am Rhein	51,8731	6,0051	not applicable		other skin disease	no	N.A.	no	N.A.	not applicable	https://www.gbif.org/occurrence/4910767143
297	not applicable	*Epidalea calamita*	24.05.23	France	Languedoc-Roussillon, 34160 Beaulieu	43,7193	4,0167	not applicable		other skin disease	no	N.A.	no	N.A.	not applicable	https://www.gbif.org/occurrence/4127067819
298	not applicable	*Epidalea calamita*	04.06.22	France	Île-de-France, Maisons-Laffitte	48,9629	2,1657	not applicable		other skin disease	no	N.A.	no	N.A.	not applicable	https://www.gbif.org/occurrence/3860127322
299	not applicable	*Epidalea calamita*	23.04.21	Spain	Communidad de Madrird, Madrid	40,7349	-3,8240	potential		BfHV1 uncertain	no	N.A.	no	N.A.	not applicable	https://www.gbif.org/occurrence/3124842448
300	not applicable	*Epidalea calamita*	11.06.21	Spain	Cataluña, Carrer Fages de Climent, Vila-sacra, Girona	42,2663	3,0167	not applicable		other skin disease	no	N.A.	no	N.A.	not applicable	https://www.gbif.org/occurrence/3307303711
301	not applicable	*Epidalea calamita*	26.05.24	France	Ciron	46,6429	1,2766	not applicable		other skin disease	no	N.A.	no	N.A.	not applicable	https://observation.org/observation/311730862/
302	not applicable	*Epidalea calamita*	26.07.24	France	Meursac	45,6495	-0,8088	not applicable		other skin disease	no	N.A.	no	N.A.	not applicable	https://observation.org/observation/321362163/
303	not applicable	*Epidalea calamita*	18.07.24	Germany	Sylt, Königshafen	55,0333	8,4080	not applicable		other skin disease	no	N.A.	no	N.A.	not applicable	https://observation.org/observation/319666840/
304	not applicable	*Epidalea calamita*	02.05.24	France	Rosnay	46,6955	1,2497	potential		BfHV1 uncertain	no	N.A.	no	N.A.	not applicable	https://observation.org/observation/307485865/
305	not applicable	*Epidalea calamita*	10.09.23	Germany	Dorsten	51,6975	6,9478	not applicable		other skin disease	no	N.A.	no	N.A.	not applicable	https://observation.org/observation/287570421/
306	not applicable	*Epidalea calamita*	16.07.23	Austria	Breitenwang	47,4893	10,7495	not applicable		other skin disease	no	N.A.	no	N.A.	not applicable	https://observation.org/observation/280827568/
307	not applicable	*Bufo bufo*	2014	Switzerland	Rothenfluh	47,4617	7,9136	first suisse record	no	1x BfHV1, assumed coordinates by	no	pos.	no	N.A.	positive	Orrigi et al. 2018
308	not applicable	*Bufo bufo*	2015	Switzerland	Rothenfluh	47,4617	7,9136	first suisse record	no	3x BfHV1, assumed coordinates b	no	pos.	no	N.A.	positive	Orrigi et al. 2018
309	not applicable	*Bufo bufo*	2016	Switzerland	Eichbuehl	47,3822	8,4735	first suisse record	no	1x BfHV1, assumed coordinates by	no	pos.	no	N.A.	positive	Orrigi et al. 2018
310	not applicable	*Bufo bufo*	2017	Switzerland	Forch	47,3246	8,6534	first suisse record	no	13x BfHV1, assumed coordinates b	no	pos.	no	N.A.	positive	Orrigi et al. 2018
311	not applicable	*Bufo bufo*	2017	Switzerland	Combacervey	47,0021	6,9357	first suisse record	no	19x BfHV1, assumed coordinates b	no	pos.	no	N.A.	positive	Orrigi et al. 2018
312	not applicable	*Bufo spinosus*	29.11.19	Portugal	Aveiro, Calvão	40,4884	-8,6914	potential	yes		no	N.A.	no	N.A.	suspicious	https://www.gbif.org/occurrence/3124692903
313	not applicable	*Bufo spinosus*	16.07.23	Portugal	Portalegre, Avis	39,0567	-7,9118	not applicable	yes	other skin disease	no	N.A.	no	N.A.	not applicable	https://www.gbif.org/occurrence/4162556501
314	not applicable	*Bufo spinosus*	23.04.24	Portugal	Braga, Estrada do sameiro	41,5506	-8,3763	potential	yes		no	N.A.	no	N.A.	suspicious	https://www.gbif.org/occurrence/4872096119
315	not applicable	*Bufo spinosus*	12.02.24	France	Provence-Alpes-Côte d'Azur, Caromb	44,1175	5,1174	potential	yes	female, flank?	no	N.A.	no	N.A.	suspicious	https://www.gbif.org/occurrence/4522729261
316	not applicable	*Bufo spinosus*	16.02.23	France	Pays de la Loire, Malville	47,3390	-1,8149	potential	yes		no	N.A.	no	N.A.	suspicious	https://www.gbif.org/occurrence/4039372553
317	not applicable	*Bufo spinosus*	25.09.21	France	Bretagne, Quimper	48,0027	-4,0979	potential	yes		no	N.A.	no	N.A.	suspicious	https://www.gbif.org/occurrence/3384350204
318	not applicable	*Bufo spinosus*	28.11.23	Portugal	Municipio do Óbidos	39,3745	-9,2175	potential	yes		no	N.A.	no	N.A.	suspicious	https://www.gbif.org/occurrence/4453968942
319	not applicable	*Bufo spinosus*	19.09.23	Portugal	Braga, Pereira	41,4949	-8,6446	potential	yes		no	N.A.	no	N.A.	suspicious	https://www.gbif.org/occurrence/4413702090
320	not applicable	*Bufo spinosus*	14.08.20	Portugal	Leiria, Reguengo do Fetal	39,6500	-8,7797	potential	yes		no	N.A.	no	N.A.	suspicious	https://www.gbif.org/occurrence/2850834779

321	not applicable	*Bufo spinosus*	11.12.17	Portugal	Santarém, Ourém	39,6533	-8,6521	potential	yes	BfHV1 uncertain	no	N.A.	no	N.A.	suspicious	https://www.gbif.org/occurrence/2611303754
322	not applicable	*Bufo spinosus*	01.05.21	Portugal	Lisbon, Lamas e Cercal	39,1794	-9,0544	potential	yes		no	N.A.	no	N.A.	suspicious	https://www.gbif.org/occurrence/3112390141
323	not applicable	*Bufo spinosus*	27.04.24	France	Aquitaine, Tarnos	43,5597	-1,4953	potential	yes		no	N.A.	no	N.A.	suspicious	https://www.gbif.org/occurrence/4852452139
324	not applicable	*Bufo spinosus*	07.10.24	France	Provence-Alpes-Côte d'Azur,Gémenos	43,3130	5,6647	potential	yes		no	N.A.	no	N.A.	suspicious	https://www.gbif.org/occurrence/4952595454
325	not applicable	*Bufo spinosus*	05.09.20	France	Provence-Alpes-Côte d'Azur, Lucerame	43,9009	7,3665	potential	yes		no	N.A.	no	N.A.	suspicious	https://www.gbif.org/occurrence/2862555808
326	not applicable	*Bufo spinosus*	19.05.24	France	Basse-Normandie, Fleuré	48,6615	-0,0532	potential	yes		no	N.A.	no	N.A.	suspicious	https://www.gbif.org/occurrence/4872330388
327	not applicable	*Bufo spinosus*	14.02.24	France	Midi-Pyrénées, Ramonville-Saint-Agne	43,5439	1,4627	potential	yes	nose parts affected	no	N.A.	no	N.A.	suspicious	https://www.gbif.org/occurrence/4872449796
328	not applicable	*Bufo spinosus*	03.01.17	France	Midi-Pyrénées, Béraut	43,9182	0,4073	potential	yes		no	N.A.	no	N.A.	suspicious	https://www.gbif.org/occurrence/1453326235
329	not applicable	*Bufo spinosus*	25.02.22	France	Provence-Alpes-Côte d'Azur,Riez	43,8137	6,0636	potential	yes		no	N.A.	no	N.A.	suspicious	https://www.gbif.org/occurrence/3802797945
330	not applicable	*Bufo spinosus*	02.09.20	France	Aquitaine, Lège-Cap-Ferret	44,7971	-1,2285	potential	yes		no	N.A.	no	N.A.	suspicious	https://www.gbif.org/occurrence/2862649325
331	not applicable	*Bufo spinosus*	19.11.24	France	Pays de la Loire, Notre-Dame-des-Landes	47,3620	-1,7117	potential	yes		no	N.A.	no	N.A.	suspicious	https://www.gbif.org/occurrence/4994496090
332	not applicable	*Bufo spinosus*	09.03.19	France	Midi-Pyrénées, Hautes-Pyrénées	43,2142	0,4064	potential	yes		no	N.A.	no	N.A.	suspicious	https://www.gbif.org/occurrence/2244254672
333	not applicable	*Bufo spinosus*	15.05.21	France	Bretagne, Hénon	48,3632	-2,6309	potential	yes		no	N.A.	no	N.A.	suspicious	https://www.gbif.org/occurrence/3117780870
334	not applicable	*Bufo spinosus*	30.03.23	France	Bretagne, Logonna-Daoulas	48,3172	-4,2934	potential	yes	BfHV1 uncertain	no	N.A.	no	N.A.	not applicable	https://www.gbif.org/occurrence/4076357238
335	not applicable	*Bufo spinosus*	20.05.20	France	Centre, Marmagne	47,0874	2,3030	potential	yes	BfHV1 uncertain	no	N.A.	no	N.A.	suspicious	https://www.gbif.org/occurrence/2802726652
336	not applicable	*Bufo spinosus*	13.07.21	France	Aquitaine, Arcachon	44,6535	-1,1796	potential	yes		no	N.A.	no	N.A.	suspicious	https://www.gbif.org/occurrence/4034695189
337	not applicable	*Bufo spinosus*	05.10.20	France	Languedoc-Roussillon, Grabels	43,6477	3,7975	potential	yes		no	N.A.	no	N.A.	suspicious	https://www.gbif.org/occurrence/2898318206
338	not applicable	*Bufo spinosus*	25.06.16	Spain	Comunidad Foral de Navarra, Gorriti	43,0497	-1,9554			other skin disease	no	N.A.	no	N.A.	not applicable	https://www.gbif.org/occurrence/3090868305
339	not applicable	*Bufo spinosus*	27.08.19	France	Bretagne, Hennebont	47,8057	-3,2735	potential	yes		no	N.A.	no	N.A.	suspicious	https://www.gbif.org/occurrence/2397586447
340	not applicable	*Bufo spinosus*	08.06.24	Spain	Galicia, Pantón	42,5155	-7,6154	potential	yes		no	N.A.	no	N.A.	suspicious	https://www.gbif.org/occurrence/4952548500
341	not applicable	*Bufo spinosus*	29.09.19	France	Pays de la Loire, La Roche-sur-Yon	46,6966	-1,4113	potential	yes		no	N.A.	no	N.A.	suspicious	https://www.gbif.org/occurrence/3067687652
342	not applicable	*Bufo spinosus*	28.04.22	France	Provence-Alpes-Côte d'Azur, Allauch	43,3316	5,4851	potential	yes	BfHV1 uncertain	no	N.A.	no	N.A.	not applicable	https://www.gbif.org/occurrence/3764326271
343	not applicable	*Bufo spinosus*	21.02.23	Portugal	Porto, São Pedro	41,2874	-8,6130	potential	yes		no	N.A.	no	N.A.	suspicious	https://www.gbif.org/occurrence/4039447993
344	not applicable	*Bufo spinosus*	02.06.20	Spain	Principado de Asturias, Asturias	43,5591	-6,6141			other skin disease	no	N.A.	no	N.A.	not applicable	https://www.gbif.org/occurrence/2634101075
345	not applicable	*Bufo spinosus*	23.02.21	France	Languedoc-Roussillon, Combaillaux	43,6693	3,7743	potential	yes	BfHV1 uncertain	no	N.A.	no	N.A.	suspicious	https://www.gbif.org/occurrence/3330421397
346	not applicable	*Bufo spinosus*	18.02.22	Portugal	Leiria, Porto de Mós	39,4964	-8,8742	potential	yes		no	N.A.	no	N.A.	suspicious	https://www.gbif.org/occurrence/3747008871
347	not applicable	*Bufo spinosus*	22.10.22	France	Aquitaine	43,4071	-1,5555	potential	yes		no	N.A.	no	N.A.	suspicious	https://www.gbif.org/occurrence/3947619418
348	not applicable	*Bufo spinosus*	09.05.24	France	Midi-Pyrénées, Haute-Garonne	43,1808	0,6479	potential	yes		no	N.A.	no	N.A.	suspicious	https://www.gbif.org/occurrence/4863780290
349	not applicable	*Bufo spinosus*	02.10.22	France	Midi-Pyrénées, Ordan-Larroque	43,6735	0,5042	potential	yes	nose parts affected	no	N.A.	no	N.A.	suspicious	https://www.gbif.org/occurrence/3923250491
350	not applicable	*Bufo spinosus*	20.10.24	Spain	Piloña	43,4100	-5,3432	potential	yes	BfHV1 uncertain	no	N.A.	no	N.A.	not applicable	https://observation.org/photos/107064609
351	not applicable	*Bufo spinosus*	19.08.24	Spain	El Rasillo de Cameros	42,1954	-2,6958	potential	yes	other skin disease	no	N.A.	no	N.A.	not applicable	https://observation.org/photos/102419545
352	not applicable	*Bufo spinosus*	21.07.24	Spain	Calonge	41,8547	3,0312	potential	yes	other skin disease	no	N.A.	no	N.A.	not applicable	https://observation.org/photos/98306070
353	not applicable	*Bufo spinosus*	13.07.24	Portugal	Vila Marim	41,3496	-7,7931	potential	yes	other skin disease	no	N.A.	no	N.A.	not applicable	https://observation.org/photos/97196386
354	not applicable	*Bufo spinosus*	13.07.24	Portugal	Vila Marim	41,3496	-7,7931	potential	yes	other skin disease	no	N.A.	no	N.A.	not applicable	https://observation.org/photos/97196382
355	not applicable	*Bufo spinosus*	21.02.24	France	Hautes-Pyrénées	43,0887	-0,0599	potential	yes		no	N.A.	no	N.A.	suspicious	https://observation.org/photos/84738225
356	not applicable	*Bufo spinosus*	24.08.23	Spain	Monreal	42,7001	-1,5026	potential	yes	other skin disease	no	N.A.	no	N.A.	not applicable	https://observation.org/photos/75899266
357	not applicable	*Bufo spinosus*	18.07.23	France	Carcans	45,0839	-1,1835	potential	yes		no	N.A.	no	N.A.	suspicious	https://observation.org/photos/72440634
358	not applicable	*Bufo spinosus*	08.06.23	Spain	Camaleño	43,1497	-4,6740	potential	yes	other skin disease	no	N.A.	no	N.A.	not applicable	https://observation.org/photos/70673076
359	not applicable	*Bufo spinosus*	18.10.22	France	Masclat	44,8152	1,3642	potential	yes	BfHV1 uncertain	no	N.A.	no	N.A.	not applicable	https://observation.org/photos/59678808
360	not applicable	*Bufo spinosus*	22.09.22	France	Esparron-de-Verdon	43,7543	5,9478	potential	yes		no	N.A.	no	N.A.	suspicious	https://observation.org/photos/58323779
361	not applicable	*Bufo spinosus*	24.09.16	France	Mas-de-Londres	43,7574	3,7482	potential	yes	BfHV1 uncertain	no	N.A.	no	N.A.	not applicable	https://observation.org/photos/12027401
362	not applicable	*Bufo spinosus*	12.08.12	Spain	Mura	41,6995	1,9765	potential	yes	other skin disease	no	N.A.	no	N.A.	not applicable	https://observation.org/photos/79775552
363	not applicable	*Bufo spinosus*	18.05.12	Spain	Riec-sur-Bélon	47,8138	-3,7153	potential	yes	BfHV1 uncertain	no	N.A.	no	N.A.	not applicable	https://observation.org/photos/109629544
364	not applicable	*Bufo spinosus*	19.07.24	France	Saint-Gaudens	43,1268	0,7164	potential	yes	BfHV1 uncertain	no	N.A.	no	N.A.	not applicable	https://observation.org/photos/98122551

Supplementary Table S2: Blastn results of each BfHV1 sequence obtained in this study. GenBank accession as reference for comparison.

Sample ID	closest hit accession	query cover	percent identity
DBEK1	NC_040681.1	100%	99.48 %
Wi23Ek3	NC_040681.1	100%	99.20 %
Ek4WS	NC_040681.1	100%	100%
TNP23EK3	NC_040681.1	100%	100%
SpEk2290322	NC_040681.1	100%	100%
Ek2Kammerstein	MT975973.1	100%	100%
Irrel24322	NC_040681.1	100%	100%

Supplementary Fig. S1: Gel electrophoresis results from end-point PCR of tested skin samples (in duplicates). Sample numbers correspond to Id's in Supplementary Table S1. ID 1-16, 20, 22, 24, 25, 28, 29, 34 and 36 show positive amplification of BfHV1 target gene (L= Ladder, ID 46= negative control, digital image taken with Alpha Imager 2.0.0.9, Alpha Innotec).

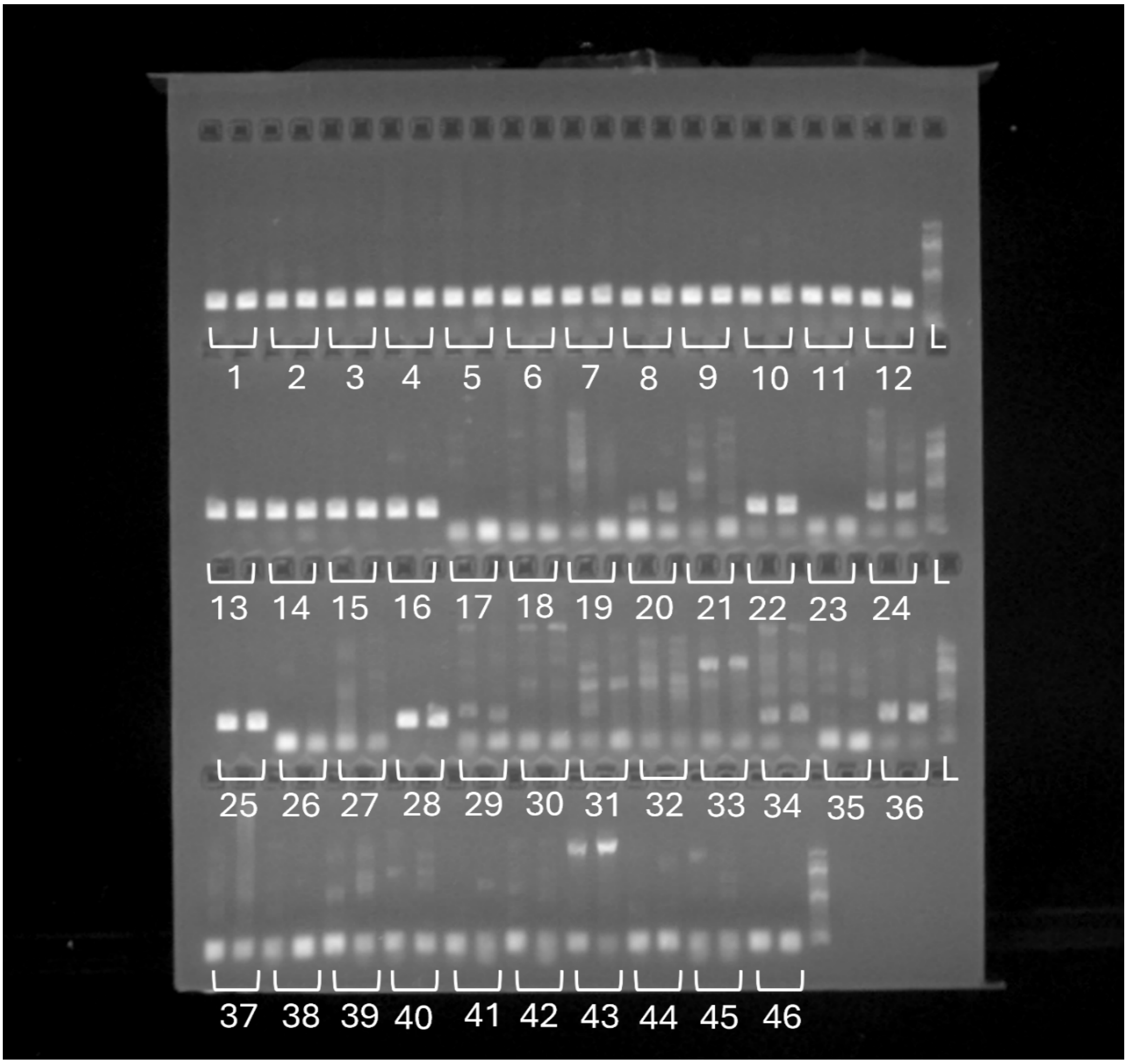

Supplementary Fig. S2: Consensus tree of samples processed in this study (#) including the two best hits from Blastn as reference (+) and two sequences of Ranid Herpes Virus (*) as outgroups (Genebank accession YP656727.1, ABG25576.1). Bootstrap values are shown as percentages (Details see text).

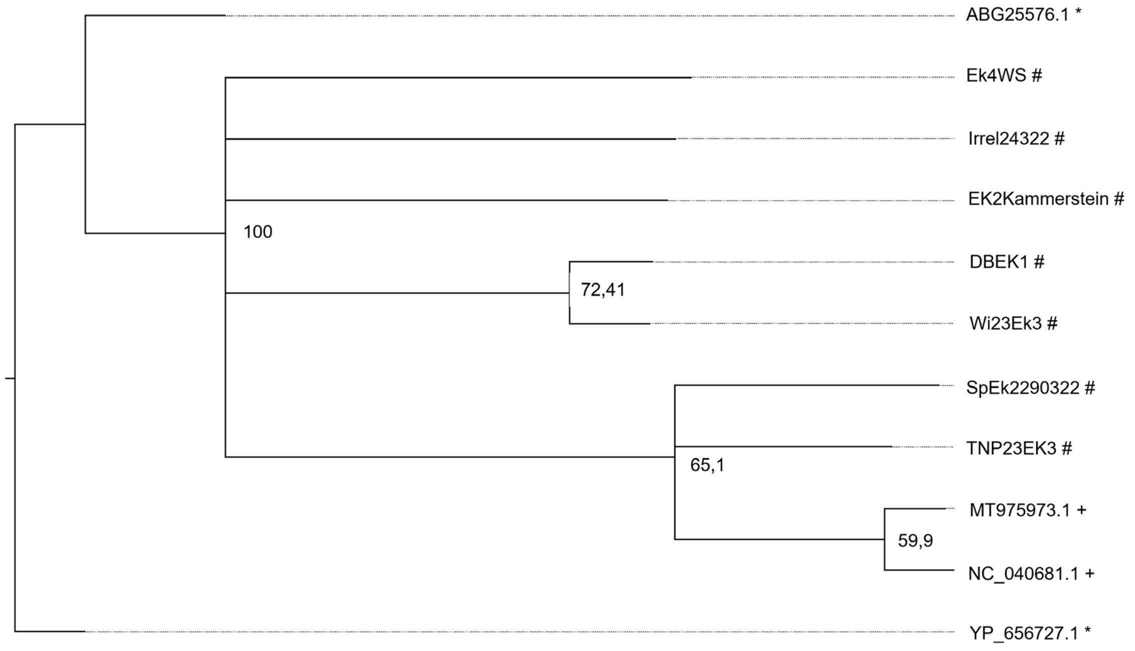

Supplementary Fig S3: Observations of *Bufo bufo* with macroskopic skin anomalies characteristic for BfHV1. A, B: Kammerstein locality, BfHV1 positive PCR results (ID 1-5, photo: H. Albrecht); C: Suspected individual from Dürrbachtal, Bavaria (ID 52, Photo: U. Martin); D: Suspected indiviudal from Waldkrailburg, Bavaria (ID 50, Photo: A. Zahn); E: Suspected individual from München, Bavaria (ID 48, Photo: T. Dürst); F: Individual showing no macroscopic skin anomalies for comparison (no ID, Photo: J. Henn; for all IDs see "BfHV1 records").

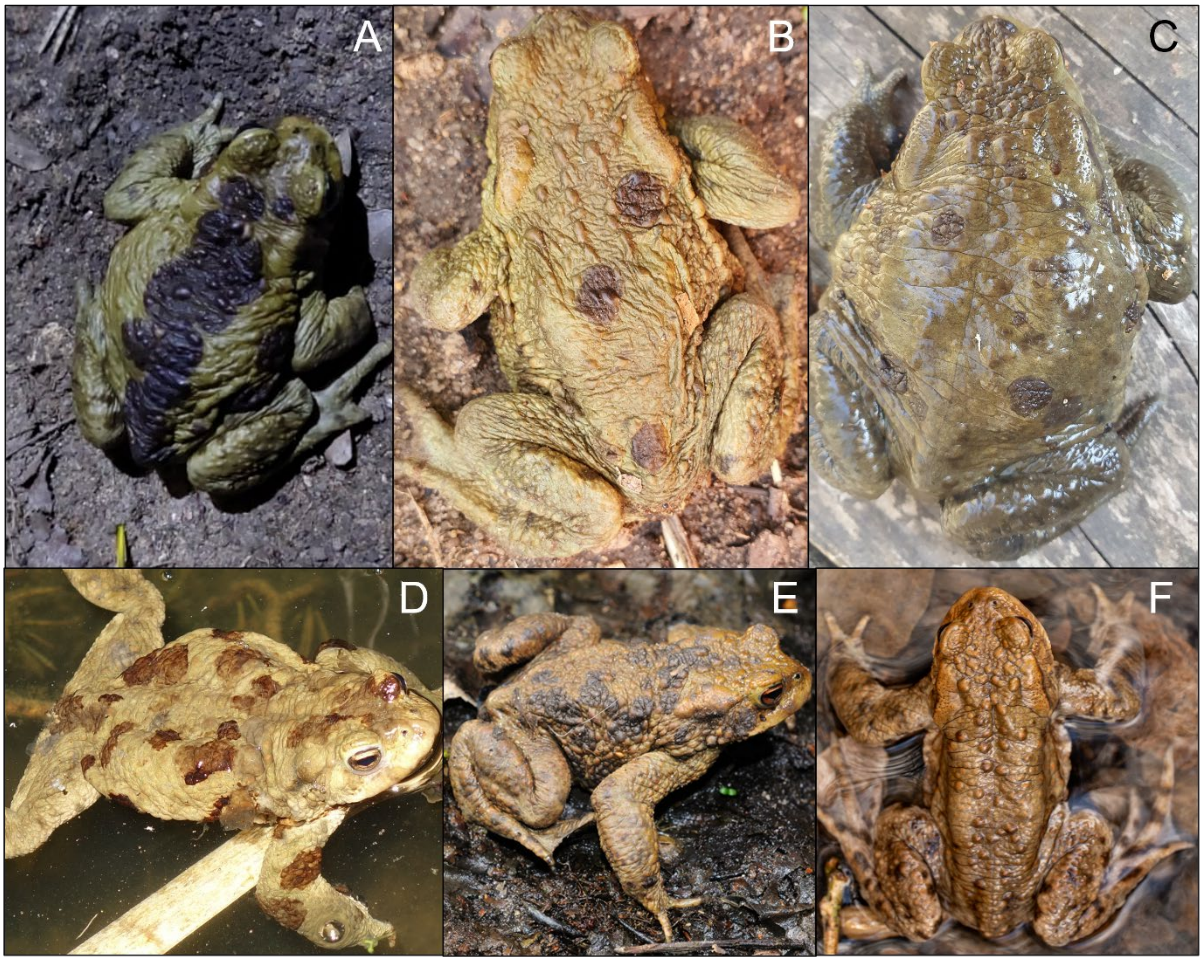

Supplementary Fig. S4: Detailed view on skin lesions on right hand (A) and left foot (B) from an adult female B. bufo (ID 25, "BfHV1 records"). Small ulcerations with dark atrium have not been reported previously for this pathogen. Swabs were collected from lesion surface.

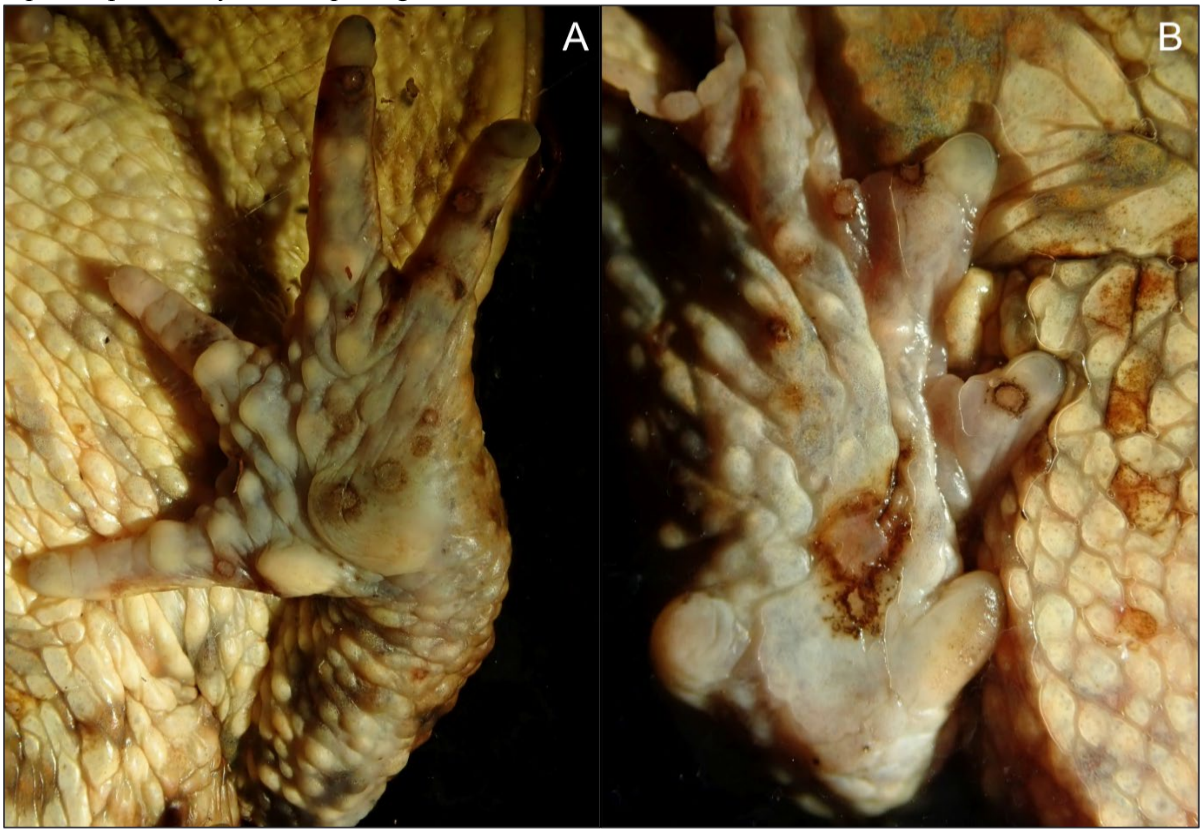

Supplementary Fig. S5: Histological slides from a preserved B. bufo from Senningerberg, Luxemburg, with clinical signs of BfHV1 infection. A: healthy skin section (100x magnification); B-D: details of diseased skin sections (B: 100x magnification, C, D: 400x magnification). HK = parakeratotic hyperkeratosis, pigment deposits, and serocellular crusts. HP = irregular epidermal hyperplasia with marked thickening of the epidermis due to an increased number of epidermal cell layers and rete ridge formation. Stars depict suspected groups of epidermocytes with intranuclear inclusion bodies, arrow depicts exocytosis of granulocytes.

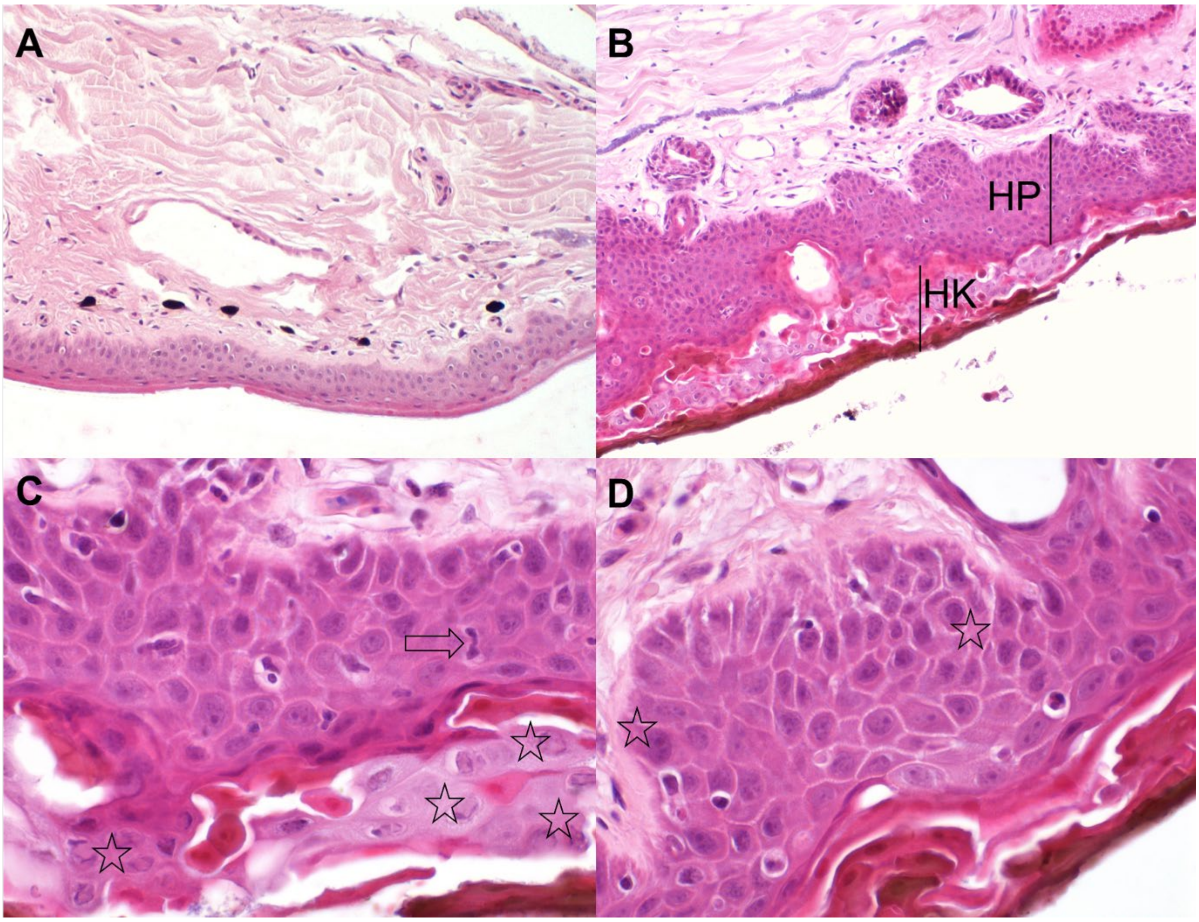

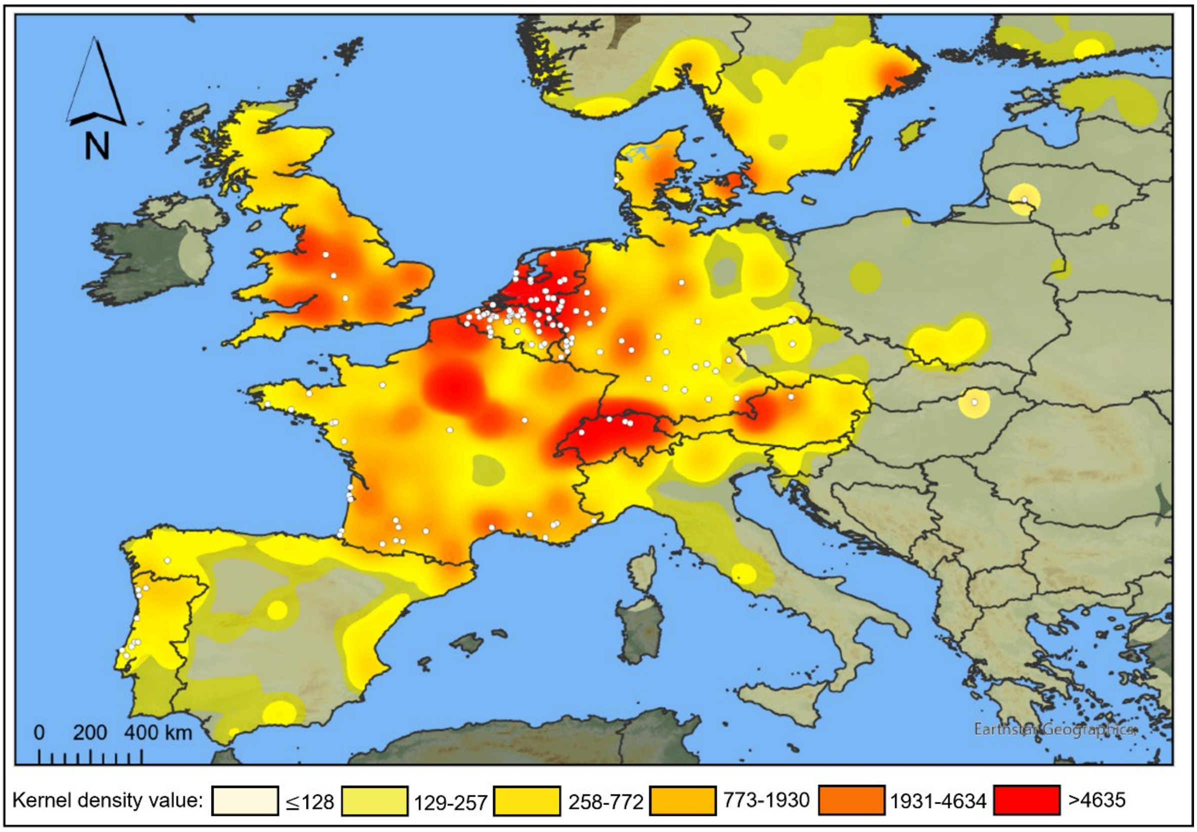

Supplementary Fig. S6. Spatial extent of *B. bufo* and *B. spinosus* citizen science records extracted from GBIF across Europe shown as a heatmap (WGS 1984). White dots correspond to suspicious cases identified in included photo databases as well as molecular and histological records (Details see text).
